# JMJD4-demethylated RIG-I prevents hepatic steatosis and carcinogenesis

**DOI:** 10.1186/s13045-022-01381-6

**Published:** 2022-11-04

**Authors:** Zhenyang Li, Ye Zhou, Kaiwei Jia, Yingyun Yang, Liyuan Zhang, Suyuan Wang, Yue Dong, Mu Wang, Yunhui Li, Shan Lu, Wannian Zhang, Luxin Zhang, Yiwen Fan, Dingji Zhang, Nan Li, Yizhi Yu, Xuetao Cao, Jin Hou

**Affiliations:** 1 National Key Laboratory of Medical Immunology and Institute of Immunology, Second Military Medical University, 800 Xiangyin Road, Shanghai, 200433 China; 2 Center for Immunotherapy, Chinese Academy of Medical Sciences, Beijing, 100005 China

**Keywords:** Hepatocarcinogenesis, Steatosis, HCC progenitor cell, RIG-I, Methylation

## Abstract

**Background:**

Hepatocarcinogenesis is driven by necroinflammation or metabolic disorders, and the underlying mechanisms remain largely elusive. We previously found that retinoic acid-inducible gene-I (RIG-I), a sensor for recognizing RNA virus in innate immune cells, is mainly expressed by parenchymal hepatocytes in the liver. However, its roles in hepatocarcinogenesis are unknown, which is intensively investigated in this study.

**Methods:**

DEN-induced necroinflammation-driven hepatocarcinogenesis and STAM NASH-hepatocarcinogenesis were carried out in hepatocyte-specific RIG-I knockout mice. The post-translational modification of RIG-I was determined by mass spectrometry, and specific antibodies against methylated lysine sites and the RIG-I lysine mutant mice were constructed to identify the functions of RIG-I methylation.

**Results:**

We interestingly found that DEN-induced hepatocarcinogenesis was enhanced, while NASH-induced hepatocarcinogenesis was suppressed by hepatocyte-specific RIG-I deficiency. Further, IL-6 decreased RIG-I expression in HCC progenitor cells (HcPCs), which then viciously promoted IL-6 effector signaling and drove HcPCs to fully established HCC. RIG-I expression was increased by HFD, which then enhanced cholesterol synthesis and steatosis, and the in-turn NASH and NASH-induced hepatocarcinogenesis. Mechanistically, RIG-I was constitutively mono-methylated at K18 and K146, and demethylase JMJD4-mediated RIG-I demethylation suppressed IL-6-STAT3 signaling. The constitutive methylated RIG-I associated with AMPKα to inhibit HMGCR phosphorylation, thus promoting HMGCR enzymatic activity and cholesterol synthesis. Clinically, RIG-I was decreased in human hepatic precancerous dysplastic nodules while increased in NAFLD livers, which were in accordance with the data in mouse models.

**Conclusions:**

Decreased RIG-I in HcPCs promotes necroinflammation-induced hepatocarcinogenesis, while increased constitutive methylated RIG-I enhances steatosis and NASH-induced hepatocarcinogenesis. JMJD4-demethylated RIG-I prevents both necroinflammation and NASH-induced hepatocarcinogenesis, which provides mechanistic insight and potential target for preventing HCC.

**Supplementary Information:**

The online version contains supplementary material available at 10.1186/s13045-022-01381-6.

## Background

Hepatocellular carcinoma (HCC) is one of the most common cancers and the leading causes of cancer-related death worldwide [1]. Hepatic inflammation, caused by viral infection, alcohol abuse, or lipid accumulation, plays critical roles in hepatocarcinogenesis [2, 3]. Hepatocyte injury and death, activation of innate immune cells in the liver, and the subsequent compensatory proliferation of hepatocytes contribute to the hepatic malignant transformation, which goes through premalignant lesions containing HCC progenitor cells (HcPCs) to the development of fully established HCC [4]. However, the underlying mechanisms of this vicious process are still largely elusive. The regulation of inflammation in hepatocarcinogenesis is therefore of great scientific and clinical interest, especially in the stage from HcPCs to established HCC.

Mechanistically, hepatocyte injury and death release damage-associated molecular patterns (DAMPs), which activate hepatic resident and recruited macrophages through Toll-like receptor (TLR) signaling. Subsequently, the production of proinflammatory cytokines such as interleukin-6 (IL-6) initiates hepatic inflammation and the following compensatory proliferation of remaining hepatocytes for repairing liver function. However, repeated hepatic injuries cause chronic inflammation, which leads to inflammation-induced hepatocarcinogenesis, and proinflammatory cytokine IL-6 and its downstream oncogenic IL-6-signal transducer and activator of transcription 3 (STAT3) pathway play the critical role in this process [5, 6]. Especially in the stages from liver cancer progenitor HcPCs to established HCC, autocrined IL-6 of HcPCs drives their progression to fully established HCC [4, 7]. Not only IL-6 production but also IL-6 effect or response is tightly regulated by multiple mechanisms, whether and how the response to IL-6 in HcPCs is dysregulated as compared to that in normal hepatocytes are still unknown up to now.

Nonalcoholic fatty liver disease (NAFLD) is characterized by excessive hepatic lipid accumulation, including both triglyceride (TG) and cholesterol [8]. NAFLD patients progress through hepatic steatosis to nonalcoholic steatohepatitis (NASH), which is characterized by chronic liver injury, inflammation, and fibrosis. NAFLD and NASH may ultimately progress to cirrhosis and eventually HCC [9]. During these processes, hepatic lipid accumulation and steatosis are the first step, which is mediated by a set of mechanisms, including the activation of sterol-regulatory element binding proteins (SREBPs), and the expression and activation of downstream metabolic enzymes [10]. However, the regulation of hepatic lipid synthesis and their roles in steatosis progression, and the following NASH and NASH-induced hepatocarcinogenesis still need further investigation.

RIG-I is well established to be one of the critical intracellular sensors for host recognition of RNA virus infection and subsequent induction of type I interferon (IFN) production in innate immune cells. It contains a C-terminal helicase domain to recognize cytoplasmic viral RNA and two N-terminal tandem caspase-recruiting domains (CARDs) to activate downstream type I IFN production [11, 12]. Other than RIG-I expression in immune cells, we previously found that RIG-I expression was mainly located in parenchymal hepatocytes in the liver, but not in mesenchymal cells [13], suggesting the potential roles of RIG-I in liver functions and diseases. However, the roles of RIG-I in hepatic metabolism, steatosis, and carcinogenesis are still unknown, especially in the different stages of hepatocarcinogenesis, such as steatosis, liver injury, inflammation, compensatory proliferation, liver cancer progenitor HcPCs, and eventually established HCC.

The function and stability of proteins are strictly controlled by multiple post-translational modifications (PTM), such as phosphorylation, ubiquitination, acetylation, and methylation [14, 15]. For the PTM of RIG-I, phosphorylation at S8, S854, S855, T170, and T770, K63-linked ubiquitination at K99, K169, K172 and K788, K48-linked ubiquitination at K813, and acetylation at K909 have been presented to tightly regulate the function of RIG-I in recognition, activation, interaction, and degradation [16]. Moreover, as protein methylation is an important PTM manner, it has been linked to transcriptional activation or repression in histones, and to protein–protein interaction between non-histones [17, 18]. However, it is still unknown up to now whether and how RIG-I is methylated or demethylated, and the roles of methylation in the regulation of RIG-I function.

In this study, we focused on the roles of RIG-I in hepatocarcinogenesis, including the expression, modification, and function of RIG-I in different hepatocarcinogenesis mouse models as well as in human tissues, to provide mechanistic insight and potential target for preventing HCC.

## Methods

### Clinical specimens

HCC patients involved in this study are Cohort 1, 152 patients from Sun Yat-sen University (Guangzhou, China), and Cohort 2, 140 patients from Guangxi Medical University (Nanning, China), who underwent radical resection of HCC as we described previously [13]. Human normal liver tissues were obtained from distal normal liver tissue of liver hemangioma patients. Liver dysplastic nodule tissues were from the corresponding patients during surgery, and these tissues were pathologically diagnosed. Human NAFLD and NASH liver tissues were collected from NAFLD and NASH patients, respectively, undergoing resection for liver hemangioma or cyst, and NASH-HCC cancer tissues were from NASH-HCC patients during surgery. These tissues were pathologically confirmed, and the patients due to excessive alcohol consumption, drug/toxin use, or viral infection were excluded. Human normal liver tissues, dysplastic nodules, NAFLD, NASH, and NASH-HCC tissues were all obtained in Second Military Medical University (Shanghai, China). All the tissue samples in this study were collected with written informed consent from the patients, and the experiments were approved by Institute Research Ethics Committee of each research center.

### Animals

C57BL/6 mice were obtained from Joint Ventures Sipper BK Experimental Animal (Shanghai, China). *Rig-I*^*f/f*^ mouse was constructed by ViewSolid Biotech (Beijing, China) using CRISPR/Cas9 techniques as previously described [19] and then mated with Alb-Cre transgenic mouse (003574), obtained from The Jackson Laboratory (Bar Harbor, ME), to generate hepatocyte-specific deficiency. *Jmjd4*^*f/f*^ mouse, *Rig-I* K18A+K146A mutant mouse, and K18M+K146M mutant mouse were constructed by Cyagen (Suzhou, China) also using CRISPR/Cas9 strategy. *IL-6*^*−/−*^ (002650), *IL-6ra*^*f/f*^ (012944), and LyzM-Cre (004781) mice were obtained from The Jackson Laboratory. *ApoE*^*−/−*^ mouse (T001458) was from GemPharmatech (Nanjing, China). Mouse genotyping was done by PCR analysis on genomic DNA extracted from tails as previously described [20]. All animals were housed in a specific pathogen-free facility and maintained in a standard temperature- and light-controlled animal facility. All animal experiments were undertaken in accordance with the National Institute of Health Guide for the Care and Use of Laboratory Animals, with the approval of the Scientific Investigation Board of Second Military Medical University, Shanghai, China.

### Mouse models

For DEN-induced hepatocarcinogenesis model, male mice at postnatal day 15 were injected intraperitoneally with DEN (25 mg/kg), and livers were examined eight months after the initial injection. For DEN plus CCl_4_ model, male mice were injected weekly with CCl_4_ (0.5 ml/kg body weight, dissolved in olive oil at a ratio of 1:3) starting from four weeks post the initial DEN injection and lasting for 15 weeks, and were sacrificed eight weeks after the last CCl_4_ injection as described [20, 21]. For acute DEN or IL-6 administration, eight-week-old male mice were injected intraperitoneally with DEN (100 mg/kg) or IL-6 (20 μg/kg), and liver tissues were examined in the indicated time post-injection [13, 22]. For metformin administration, eight-week-old male mice were fasted overnight, refed for two hours, and injected intraperitoneally with metformin (250 mg/kg) for one hour, and liver tissues were then examined. For STAM hepatocarcinogenesis model, male mice at postnatal day two were injected subcutaneously with STZ (200 μg per mouse), and fed with HFD (D12492, Research Diets, New Brunswick, NJ) for five months starting from one-month-old [23]. For WD plus CCl_4_ hepatocarcinogenesis model, two-month-old mice were fed with WD (D09100310, Research Diets) and injected weekly with CCl_4_ (0.2 ml/kg body weight) for 24 weeks. For NASH models, one-month-old male mice were fed with MCD (A06071301B, Research Diets) for four weeks or with CD-HFD (D05010403, Research Diets) for two months; and male mice were fed with HFD for five months to generate steatosis [24].

### Statistical analysis

Data are presented as mean ± s.d. from one representative of at least three independent experiments. Statistical comparisons between experimental groups were analyzed by Student’s *t*-test, chi-square test, or one-way ANOVA in SPSS 17.0 (Chicago, IL), and *P* < 0.05 was taken to indicate statistical significance. Correlation was analyzed using Pearson’s correlation coefficient assay in SPSS 17.0 with *r* and *P* values shown. For Kaplan–Meier survival analysis of patients, log-rank test in SPSS 17.0 was used with *P* values shown.

Detailed methods for the other in vivo and in vitro experiments are described in Additional file 1.

## Results

### Decreased RIG-I expression in HcPCs is due to IL-6 stimulation in the late phase

**Fig. 1:**
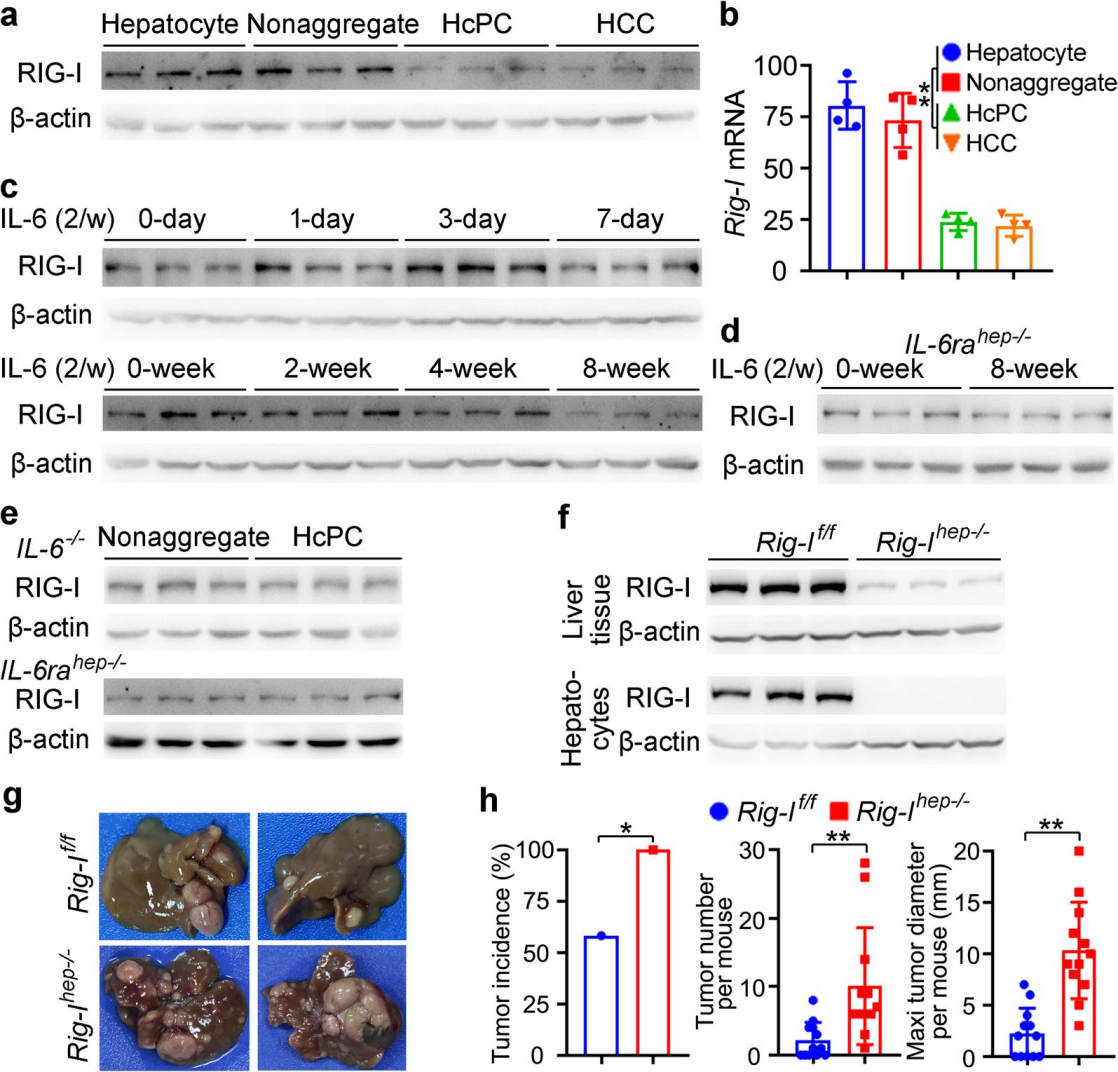
IL-6-induced RIG-I decrease in HcPCs promotes hepatocarcinogenesis. **a**, **b** RIG-I expression in isolated normal hepatocytes, nonaggregated hepatocytes and HcPCs from male mice five months post-DEN injection, and established HCC cells eight months post-DEN injection was examined by Western blot (**a**) and qRT-PCR (**b**, *n* = 4, one-way ANOVA and Tukey’s multiple comparisons test). **c** RIG-I expression was examined in male mouse livers in the indicated time periods post continuous intraperitoneal IL-6 injection twice a week. **d** RIG-I expression was examined in liver tissues of *IL-6ra*^*hep−/−*^ mice treated as in **c**. **e** RIG-I expression was examined in nonaggregate hepatocytes and HcPCs isolated from male *IL-6*^*−/−*^ or *IL-6ra*^*hep−/−*^ mice five months post-DEN injection. **f** RIG-I expression in liver tissues and isolated hepatocytes from *Rig-I*^*hep−/−*^ mice was confirmed by Western blot. **g** Representative livers of DEN-induced HCC in male *Rig-I*^*f/f*^ and *Rig-I*^*hep−/−*^ mice. **h** Tumor incidence (chi-square test), number and maximum diameter (unpaired *t*-test) in **g** were analyzed (*n* = 12). Data are shown as mean ± s.d. or photographs from one representative of three independent experiments. *P < 0.05, **P < 0.01

Chemical carcinogen DEN-induced hepatocarcinogenesis goes through premalignant liver cancer progenitor HcPCs to fully established HCC, and we first examined RIG-I expression during hepatocarcinogenesis, including normal hepatocytes, nonaggregated hepatocytes and aggregates containing HcPCs from mice five months post-DEN injection [4, 25], and established HCC cells eight months post-DEN injection. Both protein and mRNA levels of RIG-I were determined to be decreased in HcPCs and established HCC cells, while its levels in normal hepatocytes and nonaggregated hepatocytes were similar (Fig. 1a, b). Thus, RIG-I expression is decreased in the stages from premalignant HcPCs to established HCC during hepatocarcinogenesis, suggesting its potential role in the development of HcPCs.

The mechanism responsible for the decreased RIG-I in HcPCs was then investigated. As the initiated hepatocytes acquire the ability to autocrine IL-6 thus becoming HcPCs during hepatocarcinogenesis [4], and knockout of IL-6 abolishes inflammation-induced HCC [26], we analyzed whether IL-6 mediated the decrease in hepatic RIG-I. By continuous injection of IL-6 twice a week in vivo, we found that hepatic RIG-I expression was increased in the early phase (within one week) while decreased in the late phase (at two months) (Fig. 1c). In hepatocyte-specific IL-6 receptor knockout *IL-6ra*^*hep*−/−^ mice, continuous IL-6 injection failed to decrease hepatic RIG-I expression (Fig. 1d). Moreover, in *IL-6*^*−/−*^ or *IL-6ra*^*hep*−/−^ mice, RIG-I expression in isolated HcPCs five months post-DEN injection was not decreased as compared to that in nonaggregated hepatocytes (Fig. 1e). Together, although IL-6 increases hepatic RIG-I expression in the early phase, its expression is decreased in HcPCs, which is mediated by continuous IL-6 stimulation in the late phase.

### *Decreased hepatic RIG-I expression promotes DEN-induced hepatocarcinogenesis*.

To analyze the role of RIG-I decrease in hepatocarcinogenesis, we constructed hepatocyte-specific RIG-I knockout *Rig-I*^*hep*−/−^ mouse (Fig. 1f, Additional file 2: Fig. S1a), and found that DEN-induced hepatocarcinogenesis was markedly promoted by hepatic RIG-I deficiency, including increased tumor incidence, number, diameter, and shortened survival (Fig. 1g, h, Additional file 2: Fig. S1b). The DEN plus CCl_4_-induced hepatocarcinogenesis model was applied, and it was also promoted by hepatic RIG-I deficiency (Additional file 2: Fig. S1c, d). Moreover, to exclude the potential effect of RIG-I as a viral RNA sensor in innate immune cells, we generated *Rig-I*^*Mac*−/−^ mice and determined that RIG-I in macrophages could not significantly influence DEN-induced hepatocarcinogenesis (Additional file 2: Fig. S1e). Together, hepatic RIG-I deficiency promotes DEN-induced HCC, suggesting that decreased RIG-I expression in HcPCs may participate in hepatocarcinogenesis.

### Decreased RIG-I expression in liver cancer progenitor HcPCs promotes their response to IL-6, which viciously drives their progression to established HCC

**Fig. 2:**
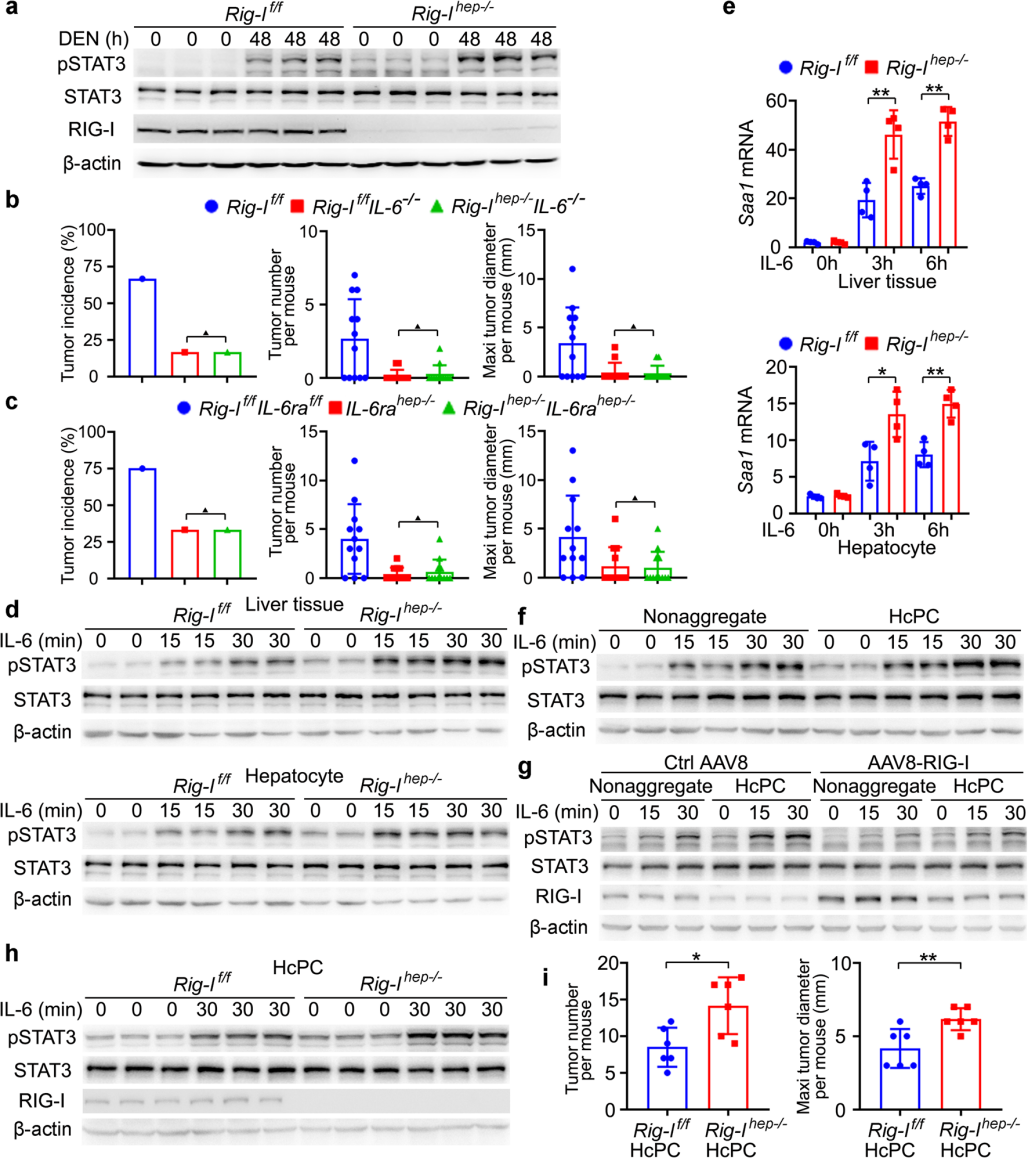
Decreased RIG-I in liver cancer progenitor HcPCs promotes their response to IL-6, which viciously drives their progression to established HCC. **a** STAT3 phosphorylation and activation were examined in liver tissues from male *Rig-I*^*f/f*^ and *Rig-I*^*hep−/−*^ mice 48 h post-DEN administration. **b** Tumor incidence (chi-square test), number and maximum diameter (unpaired *t*-test) of DEN-induced HCC in male *Rig-I*^*f/f*^*IL-6*^*−/−*^ and *Rig-I*^*hep−/−*^*IL-6*^*−/−*^ mice were analyzed (*n* = 12). **c** Tumor incidence (chi-square test), number and maximum diameter (unpaired *t*-test) of DEN-induced HCC in male *IL-6ra*^*hep−/−*^ and *Rig-I*^*hep−/−*^*IL-6ra*^*hep−/−*^ mice were analyzed (*n* = 12). **d** IL-6-induced STAT3 phosphorylation in the indicated time periods was examined in liver tissues and isolated hepatocytes from male *Rig-I*^*f/f*^ and *Rig-I*^*hep−/−*^ mice. **e** IL-6-induced *Saa1* mRNA expression in the indicated time periods was examined in liver tissues and isolated hepatocytes from male *Rig-I*^*f/f*^ and *Rig-I*^*hep−/−*^ mice (*n* = 4, unpaired *t*-test). **f** Isolated nonaggregated hepatocytes and HcPCs were stimulated with IL-6 for the indicated time periods, and STAT3 phosphorylation was examined. **g** Isolated nonaggregated hepatocytes and HcPCs from control AAV8 or AAV8-RIG-I-treated male mice were stimulated as in **f**, and STAT3 phosphorylation was examined. **h** Isolated HcPCs from male *Rig-I*^*f/f*^ and *Rig-I*^*hep−/−*^ mice were stimulated with IL-6 for 30 min, and STAT3 phosphorylation was examined. **i** Tumor number and maximum diameter were analyzed in male mice transplanted by intrasplenic injection with equal amounts of isolated HcPCs from *Rig-I*^*f/f*^ and *Rig-I*^*hep−/−*^ mice (*n* = 6, unpaired *t*-test). Data are shown as mean ± s.d. or photographs from one representative of three independent experiments. *P < 0.05, **P < 0.01, ^▲^*P* > 0.05

We next examined the underlying mechanism responsible for *Rig-I*^*hep*−/−^-promoted hepatocarcinogenesis. The DNA damage, hepatocyte apoptosis, and serum ALT and AST representing liver damage following acute DEN injection were analyzed in *Rig-I*^*hep*−/−^ mice, and DEN-induced liver damage was not influenced by hepatic RIG-I deficiency (Additional file 2: Fig. S2a–e). The DEN-induced hepatic production of proinflammatory cytokines including IL-6 and TNF-α, infiltration of leukocytes, and compensatory hepatocyte proliferation were also not significantly influenced by *Rig-I*^*hep*−/−^ (Additional file 2: Fig. S2f–h). We then performed protein phosphorylation microarray to elucidate the altered intracellular signaling in *Rig-I*^*hep−*/−^ liver following acute DEN injection, and found that STAT3 phosphorylation was the most increased by hepatic RIG-I deficiency (Additional file 2: Fig. S2i). The increased DEN-induced STAT3 phosphorylation was then confirmed in *Rig-I*^*hep*−/−^ liver (Fig. 2a), suggesting that RIG-I deficiency may enhance STAT3 activation to promote hepatocarcinogenesis.

As IL-6 effects mainly through Janus kinase 2 (JAK2)-STAT3 signaling and is the key proinflammatory cytokine driving hepatocarcinogenesis [26, 27], we examined whether hepatic RIG-I deficiency-promoted hepatocarcinogenesis was dependent on IL-6-STAT3 signaling. *Rig-I*^*hep−/−*^*IL-6*^*−/−*^ double knockout (DKO) mice and *Rig-I*^*hep−/−*^*IL-6ra*^*hep−/−*^ DKO mice were generated, respectively. Loss of IL-6 diminished DEN-induced HCC, and importantly, *Rig-I*^*hep−/−*^*IL-6*^*−/−*^ mice and *IL-6*^*−/−*^ mice displayed a similar reduced induction of HCC by DEN (Fig. 2b), suggesting that hepatic RIG-I deficiency failed to promote hepatocarcinogenesis under IL-6 deficiency. Similar results were obtained in *Rig-I*^*hep−/−*^*IL-6ra*^*hep−/−*^ DKO mice (Fig. 2c). Therefore, hepatocyte-specific RIG-I deficiency-promoted hepatocarcinogenesis is dependent on the proinflammatory cytokine IL-6.

DEN-induced production of hepatic IL-6 was not influenced by RIG-I deficiency (Additional file 2: Fig. S2f), whether hepatic RIG-I deficiency promotes hepatocarcinogenesis by enhancing IL-6-STAT3 effector signaling was then examined. We found that IL-6-induced STAT3 phosphorylation was significantly promoted by hepatic RIG-I deficiency, both upon IL-6 injection in vivo and IL-6 stimulation in primary hepatocytes in vitro (Fig. 2d). The expression of IL-6-induced downstream gene *Saa1* was also enhanced by hepatic RIG-I deficiency (Fig. 2e). Thus, hepatic RIG-I deficiency-promoted hepatocarcinogenesis is mediated by the enhanced oncogenic IL-6-STAT3 effector signaling.

Since RIG-I expression is decreased in liver cancer progenitor HcPCs and RIG-I deficiency promotes IL-6-driven hepatocarcinogenesis, we presumed that not only IL-6 was autocrined by HcPCs, but also their response to IL-6 was enhanced. IL-6-induced STAT3 activation was significantly enhanced in HcPCs as compared to that in nonaggregated hepatocytes (Fig. 2f). We also rescued RIG-I expression in HcPCs using AAV8-mediated gene delivery, and confirmed that IL-6-STAT3 signaling was compromised, to the level similar with that in nonaggregated hepatocytes of control mice (Fig. 2g). Hence, the response to IL-6 is enhanced in HcPCs, which is mediated by the decreased RIG-I expression.

To confirm that decreased RIG-I in HcPCs promotes hepatocarcinogenesis, especially in the stages from HcPCs to established HCC, we isolated CD44^+^ HcPCs from the livers of *Rig-I*^*f/f*^ and *Rig-I*^*hep−/−*^ mice five months post-DEN injection, and determined that IL-6-induced STAT3 activation was enhanced by RIG-I deficiency in HcPCs (Fig. 2h). Equal amounts of HcPCs from *Rig-I*^*f/f*^ or *Rig-I*^*hep−/−*^ were transplanted into wildtype mice, which were then injected weekly with CCl_4_ to induce liver inflammation for five months to drive the transplanted HcPCs to established HCC [4]. RIG-I deficient HcPCs generated more and larger HCC nodules as compared to those of HcPCs from *RIG-I*^*f/f*^ mice (Fig. 2i), suggesting that decreased RIG-I in HcPCs promotes their progression to HCC. Altogether, we conclude that IL-6-induced RIG-I decrease and decreased RIG-I-enhanced IL-6 response in HcPCs may cause vicious feedforward progression from premalignant HcPCs to fully established HCC.

### *RIG-I associates with STAT3 to impede JAK2-STAT3 interaction and inhibit IL-6 effector signaling*.

**Fig. 3:**
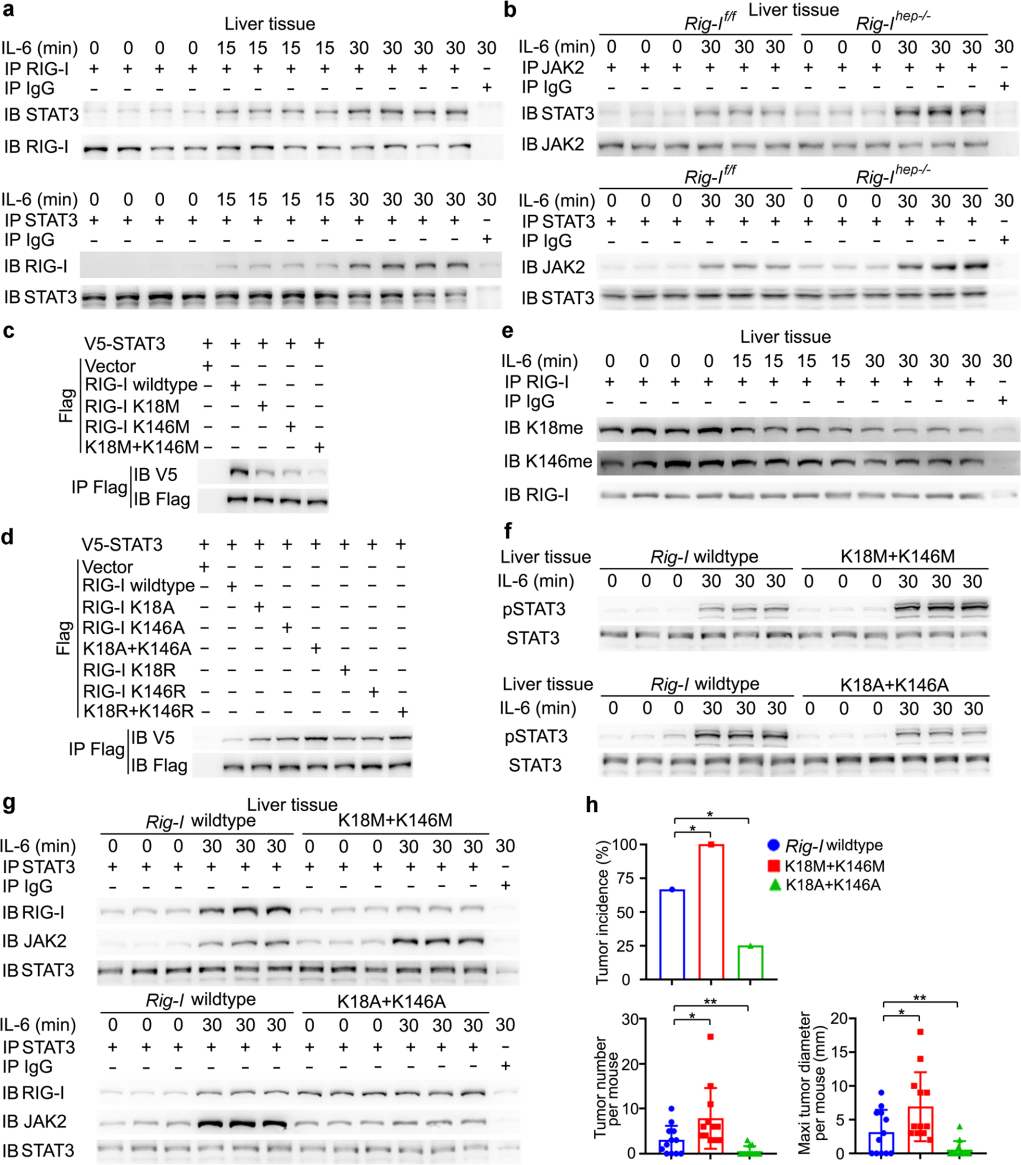
IL-6 induces RIG-I demethylation to enhance RIG-I-STAT3 association and feedback impede JAK2-STAT3 interaction. **a** RIG-I-STAT3 association induced by IL-6 was examined by immunoprecipitation in male mouse liver tissues. **b** IL-6-induced JAK2-STAT3 interaction was evaluated by immunoprecipitation in liver tissues from male *Rig-I*^*f/f*^ and *Rig-I*^*hep−/−*^ mice. **c**, **d** V5-tagged STAT3 and Flag-tagged RIG-I mutants as indicated were transfected into HHL5 hepatocyte cell line, and their association was tested by immunoprecipitation. **e** Methylated RIG-I at K18 and K146 were examined by the specific antibodies in the precipitates by total RIG-I antibody from the liver tissues upon IL-6 stimulation. **f** IL-6-induced STAT3 phosphorylation was evaluated in liver tissues from wildtype, RIG-I K18M+K146M or K18A+K146A mutant male mice upon IL-6 stimulation. **g** IL-6-induced RIG-I-STAT3 association and JAK2-STAT3 interaction were examined using immunoprecipitation in the liver tissues from male wildtype, RIG-I K18M+K146M or K18A+K146A mutant mice upon IL-6 stimulation. **h** Tumor incidence (chi-square test), number and maximum diameter (unpaired *t*-test) of DEN-induced HCC in male wildtype, RIG-I K18M+K146M or K18A+K146A mutant mice were analyzed (*n* = 12). Data are shown as mean ± s.d. or photographs from one representative of three independent experiments. *P < 0.05, **P < 0.01

The mechanism responsible for RIG-I-mediated inhibition of IL-6-STAT3 signaling was then examined. We found that IL-6 could induce the association between RIG-I and STAT3, both upon IL-6 injection in vivo and IL-6 stimulation in primary hepatocytes in vitro (Fig. 3a, Additional file 2: Fig. S3a). To determine how RIG-I binding to STAT3 suppresses STAT3 activation, we screened the kinases and regulators involved in IL-6-STAT3 signaling, and found that STAT3 could bind to JAK1, JAK2, SHP1, SHP2, and SOCS3, but not SOCS1, PIAS1, or PIAS3, upon IL-6 stimulation in the liver (Additional file 2: Fig. S3b). Among them, IL-6-induced JAK2-STAT3 interaction was significantly enhanced by hepatic RIG-I deficiency, both by IL-6 injection in vivo and IL-6 stimulation in primary hepatocytes in vitro (Fig. 3b, Additional file 2: Fig. S3c), while IL-6-induced JAK2 phosphorylation was not influenced (Additional file 2: Fig. S3d). As JAK2-phosphorylated STAT3 is the key pathway in IL-6 effects, and RIG-I deficiency failed to promote IL-6-STAT3 activation under inhibition of JAK2 (Additional file 2: Fig. S3e), hepatic RIG-I deficiency-promoted STAT3 activation is mediated by the enhanced JAK2-STAT3 interaction. Moreover, the tagged truncates of RIG-I, JAK2, and STAT3 were constructed, and both CARD domain of RIG-I and JH2 domain of JAK2 could bind the SH2-TA domain of STAT3, which contains the phosphorylation site tyrosine 705 (Additional file 2: Fig. S3f). Thus, RIG-I associates with STAT3 to impede JAK2-STAT3 interaction and inhibit IL-6 effector signaling. Together with the IL-6-induced hepatic RIG-I expression in the early phase (Fig. 1c), all these data determine that IL-6 induces RIG-I-STAT3 association and RIG-I expression to feedback inhibit JAK2-STAT3 interaction and IL-6 effector signaling in the early phase.

### *IL-6 induces the demethylation of RIG-I to enhance RIG-I-STAT3 association*.

We next examined the mechanism responsible for IL-6-induced RIG-I-STAT3 association. As protein PTM plays critical roles in protein–protein interaction, the PTM of RIG-I following IL-6 stimulation was analyzed by mass spectrometry, especially in the CARD domain which is responsible for RIG-I-STAT3 association. Methylation, acetylation, and phosphorylation of RIG-I were screened. Mono-methylation at K18, K146, and acetylation at K95, K172 were identified in untreated, while acetylation at K59, K154 were identified in IL-6-stimulated hepatocytes, and mono-methylation at K48 and phosphorylation at S8, T55 were identified in both. The mutants K48A, K59A, K95A, K154A, K172A, S8A, and T55A had no effect on the RIG-I-STAT3 association (Additional file 2: Fig. S3g). However, the mutants K18M and K146M mimicking mono-methylation of evolutionarily conserved K18 and K146 both significantly decreased RIG-I-STAT3 association, and K18M + K146M mutant abolished the association (Fig. 3c, Additional file 2: Fig. S3h, i). The K18A, K146A, K18R, K146R mutants of RIG-I mimicking demethylation were also constructed, and their association with STAT3 were significantly enhanced (Fig. 3d), suggesting that demethylation of RIG-I at both K18 and K146 may be important for RIG-I-STAT3 association. Furthermore, rabbit polyclonal antibodies specifically recognizing the K18 or K146 mono-methylated RIG-I were generated, respectively (Additional file 2: Fig. S3j, k), and IL-6-induced RIG-I demethylation at these sites was confirmed both in liver tissues in vivo and in primary hepatocytes in vitro (Fig. 3e, Additional file 2: Fig. S3l). Thus, IL-6-induced RIG-I-STAT3 association was dependent on the induced demethylation at K18 and K146 of hepatic RIG-I.

To confirm the role of demethylated RIG-I in associating and suppressing IL-6-induced STAT3 activation, we constructed the K18M+K146M mutant mouse mimicking mono-methylated RIG-I, and K18A+K146A mutant mouse mimicking demethylated RIG-I (Additional file 2: Fig. S3m). IL-6-induced hepatic STAT3 phosphorylation was significantly increased in K18M+K146M mice, while decreased in K18A+K146A mice, both by IL-6 injection in vivo and IL-6 stimulation in primary hepatocytes in vitro (Fig. 3f and Additional file 2: Fig. S3n). Similarly, hepatic RIG-I-STAT3 association was markedly decreased in K18M+K146M mice, while increased in K18A+K146A mice; IL-6-induced JAK2-STAT3 association was enhanced in K18M+K146M mice, while decreased in K18A+K146A mice (Fig. 3g). Furthermore, DEN-induced hepatocarcinogenesis was increased in K18M+K146M mice, while decreased in K18A+K146A mice (Fig. 3h). Together, we conclude that IL-6-induced RIG-I demethylation at K18 and K146 feedback associates with STAT3 and inhibits IL-6-STAT3 effector signaling.

### *Demethylase JMJD4 is responsible for IL-6-induced demethylation of RIG-I*.

**Fig. 4:**
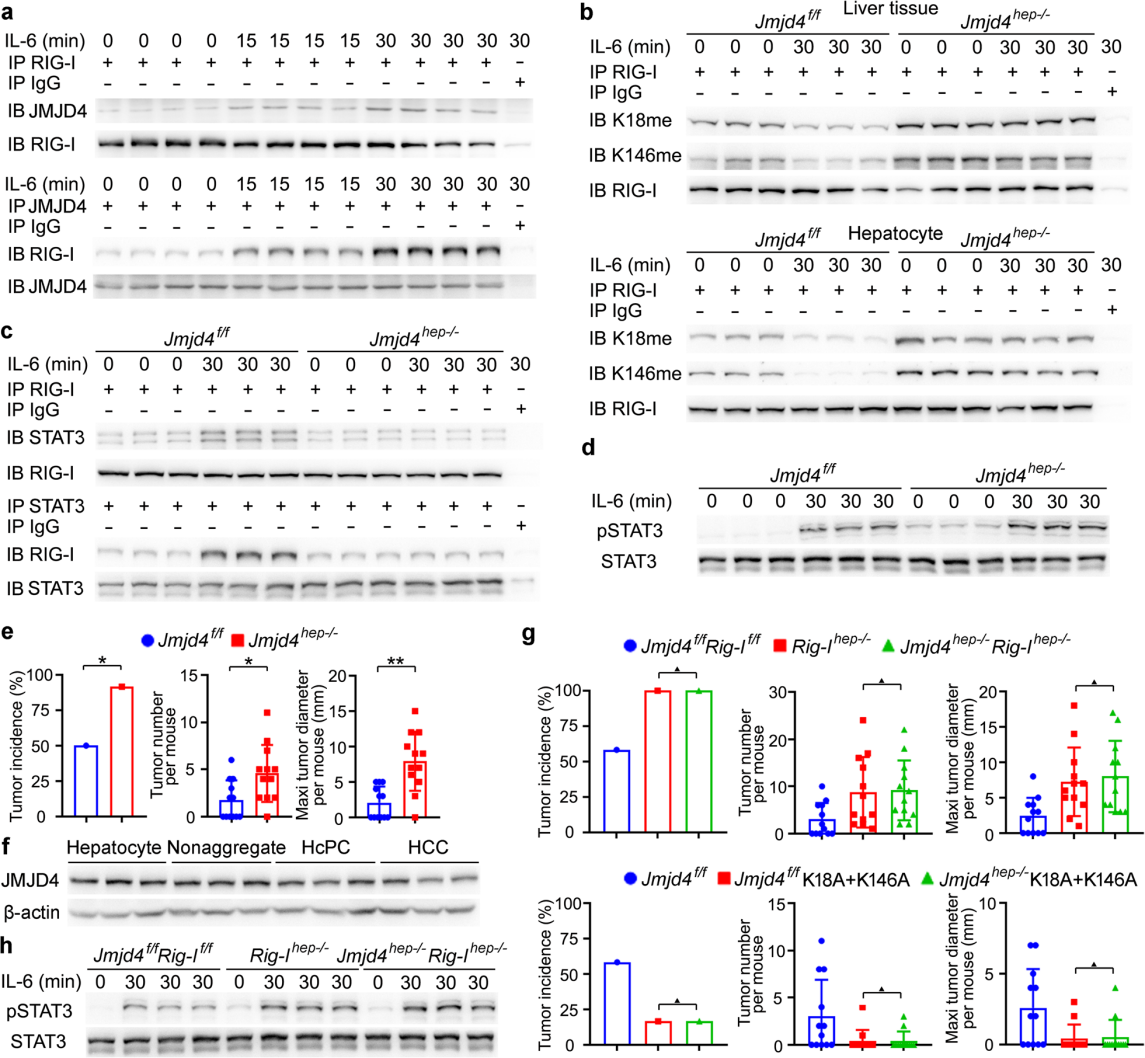
JMJD4-demethylated RIG-I inhibits DEN-induced hepatocarcinogenesis. **a** JMJD4-RIG-I association induced by IL-6 was examined by immunoprecipitation in male mouse liver tissues. **b** Methylated RIG-I at K18 and K146 upon IL-6 stimulation was examined in male *Jmjd4*^*f/f*^ and *Jmjd4*^*hep−/−*^ liver tissues and isolated primary hepatocytes as indicated. **c** IL-6-induced RIG-I-STAT3 association was evaluated by immunoprecipitation in liver tissues from male *Jmjd4*^*f/f*^ and *Jmjd4*^*hep−/−*^ mice. **d** IL-6-induced STAT3 phosphorylation was evaluated in liver tissues from male *Jmjd4*^*f/f*^ and *Jmjd4*^*hep−/−*^ mice upon IL-6 stimulation. **e** Tumor incidence (chi-square test), number and maximum diameter (unpaired *t*-test) of DEN-induced HCC in *Jmjd4*^*f/f*^ and *Jmjd4*^*hep−/−*^ mice were analyzed (*n* = 12). **f** JMJD4 expression in isolated normal hepatocytes, nonaggregated hepatocytes and HcPCs from male mice five months post-DEN injection, and established HCC cells eight months post-DEN injection was examined by Western blot. **g** Tumor incidence (chi-square test), number and maximum diameter (unpaired *t*-test) of DEN-induced HCC in male *Jmjd4*^*f/f*^*Rig-I*^*f/f*^, *Rig-I*^*hep−/−*^ and *Jmjd4*^*hep−/−*^*Rig-I*^*hep−/−*^ mice, or in male *Jmjd4*^*f/f*^, *Jmjd4*^*f/f*^ RIG-I K18A+K146A and *Jmjd4*^*hep−/−*^ RIG-I K18A+K146A mice as indicated were analyzed (*n* = 12). **h** IL-6-induced STAT3 phosphorylation was evaluated in liver tissues from male *Jmjd4*^*f/f*^*Rig-I*^*f/f*^, *Rig-I*^*hep−/−*^ and *Jmjd4*^*hep−/−*^*Rig-I*^*hep−/−*^ mice upon IL-6 stimulation. Data are shown as mean ± s.d. or photographs from one representative of three independent experiments. *P < 0.05, **P < 0.01, ^▲^*P* > 0.05

The mechanism responsible for IL-6-induced RIG-I demethylation was then investigated. We immunoprecipitated Flag-tagged RIG-I from lysates of IL-6-stimulated HHL5 hepatocyte cell line, then used mass spectrometry to identify RIG-I-associated proteins, and selected JMJD4 as the candidate because of its demethylase activity (Additional file 2: Fig. S4a). The IL-6-induced JMJD4-RIG-I association was confirmed by immunoprecipitation (Fig. 4a). The tagged truncates were constructed, and JMJC demethylase catalytic domain of JMJD4 also associated with the CARD domain of RIG-I, where K18 and K146 locate (Additional file 2: Fig. S4b). Thus, demethylase JMJD4 associates with RIG-I following IL-6 stimulation, which may participate in the IL-6-induced RIG-I demethylation.

We next constructed hepatocyte-specific JMJD4 knockout mouse (Additional file 2: Fig. S4c, d), and determined that IL-6-induced hepatic RIG-I demethylation at K18 and K146 was abolished by JMJD4 deficiency, both by IL-6 injection in vivo and IL-6 stimulation in primary hepatocytes in vitro (Fig. 4b). IL-6-induced RIG-I-STAT3 association was markedly decreased while STAT3 phosphorylation was enhanced by hepatic JMJD4 deficiency (Fig. 4c, d). Together, these data determine that IL-6 induces JMJD4-erased methylation of RIG-I to enhance RIG-I-STAT3 association and feedback inhibit IL-6-STAT3 signaling.

### *JMJD4 and RIG-I co-operatively suppress DEN-induced hepatocarcinogenesis*.

As JMJD4-erased methylation of RIG-I feedback suppresses IL-6-STAT3 effector signaling, we further examined the role of JMJD4 in DEN-induced hepatocarcinogenesis. Hepatocyte-specific JMJD4 knockout significantly promoted both DEN and DEN plus CCl_4_-induced hepatocarcinogenesis (Fig. 4e, Additional file 2: Fig. S4e), which is similar to those in *Rig-I*^*hep−/−*^ mice. JMJD4 expression was also analyzed in mouse HcPCs and HCC cells, and its expression moderately decreased as compared to that in normal hepatocytes and nonaggregates (Fig. 4f, Additional file 2: Fig. S4f). Together with the data that the markedly decreased RIG-I in HcPCs promotes hepatocarcinogenesis, this potentially decreased JMJD4 may further enhance IL-6 response and the corresponding hepatocarcinogenesis.

The hepatocyte-specific JMJD4 and RIG-I DKO mouse and *Jmjd4*^*hep−/−*^ plus RIG-I K18A+K146A mouse were then generated, respectively, and we found that the DEN-induced hepatocarcinogenesis in *Jmjd4*^*hep−/−*^*Rig-I*^*hep−/−*^ mice was similar to that in *Rig-I*^*hep−/−*^ mice, and hepatocarcinogenesis in *Jmjd4*^*hep−/−*^ plus RIG-I K18A+K146A mice was similar to that in *Jmjd4*^*f/f*^ plus RIG-I K18A+K146A mouse (Fig. 4g), suggesting that JMJD4 deficiency-promoted hepatocarcinogenesis is dependent on RIG-I and its demethylation. Moreover, IL-6-induced hepatic STAT3 phosphorylation was enhanced in *Jmjd4*^*hep−/−*^*Rig-I*^*hep−/−*^ mice, which was similar to that in *Rig-I*^*hep−/−*^ mice (Fig. 4h). Thus, IL-6-induced hepatic JMJD4-RIG-I association co-operatively feedback suppresses IL-6-STAT3 effector signaling and DEN-induced hepatocarcinogenesis. Altogether, we conclude that decreased RIG-I in liver cancer progenitor HcPCs promotes their response to IL-6, which drives the progression from HcPCs to fully established HCC in the DEN model mimicking necroinflammation-induced hepatocarcinogenesis.

### Opposite to DEN model, *Rig-I*^*hep−/−*^ suppressed NASH-induced HCC

**Fig. 5:**
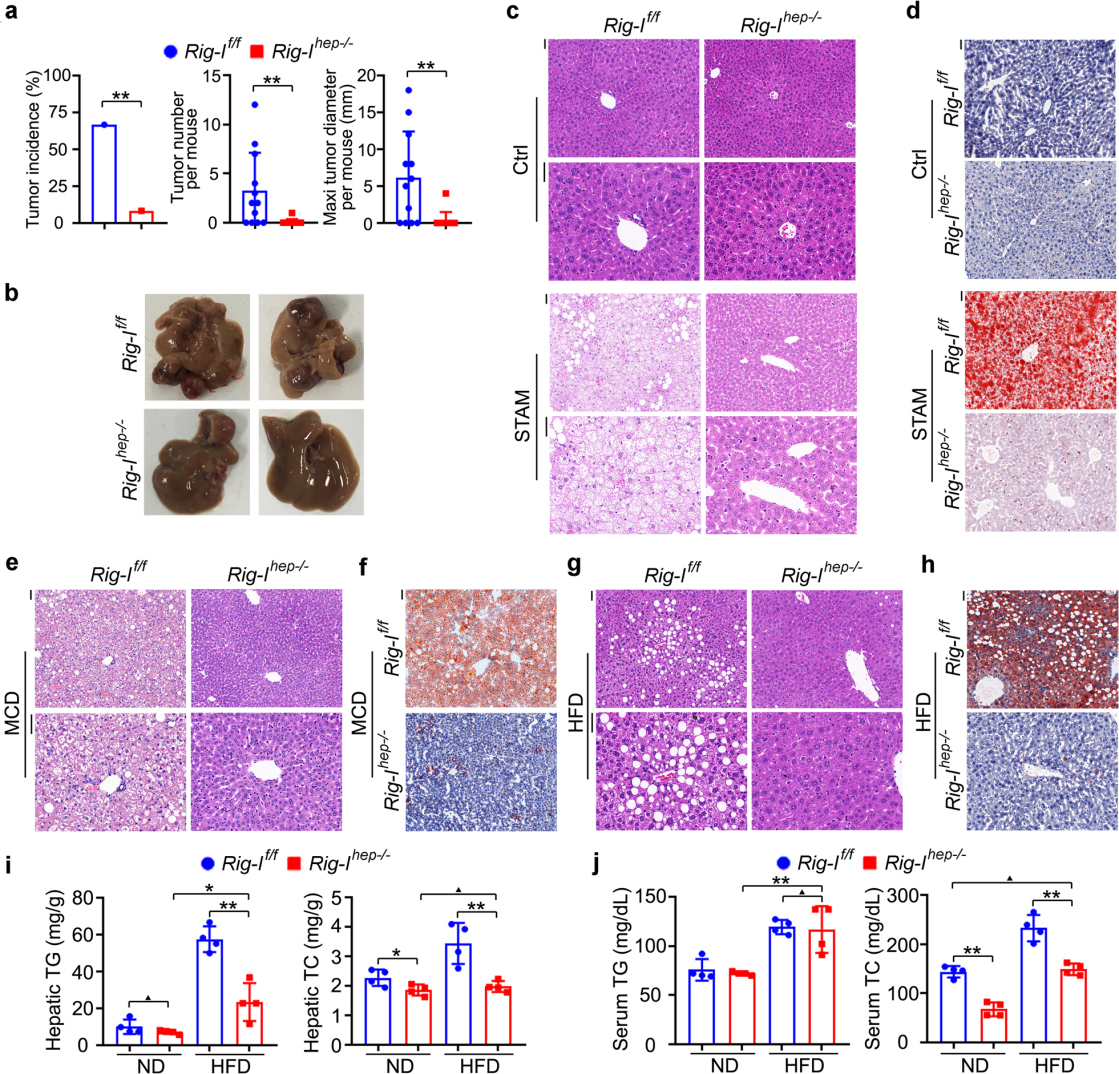
Hepatocyte-specific RIG-I deficiency abolishes steatosis, and the following NASH, and NASH-induced hepatocarcinogenesis. **a** Tumor incidence (chi-square test), number and maximum diameter (unpaired *t*-test) of STAM HCC in male *Rig-I*^*f/f*^ and *Rig-I*^*hep−/−*^ mice were analyzed (*n* = 12). **b** Representative livers of STAM HCC in male *Rig-I*^*f/f*^ and *Rig-I*^*hep−/−*^ mice. **c**, **d** HE (**c**) and oil red O (**d**) staining were analyzed in nontumor liver tissues of STAM model in male *Rig-I*^*f/f*^ and *Rig-I*^*hep−/−*^ mice. Scale bars: 20 μm. **e**, **f** HE (**e**) and oil red O (**f**) staining were analyzed in liver tissues of MCD model in male *Rig-I*^*f/f*^ and *Rig-I*^*hep−/−*^ mice. Scale bars: 20 μm. **g**, **h** HE (**g**) and oil red O (**h**) staining were analyzed in liver tissues of HFD model in male *Rig-I*^*f/f*^ and *Rig-I*^*hep−/−*^ mice. Scale bars: 20 μm. **i**, **j** Hepatic TG and TC (**i**), and serum TG and TC (**j**) were examined in HFD-treated male *Rig-I*^*f/f*^ and *Rig-I*^*hep−/−*^ mice (*n* = 4, unpaired *t*-test). Data are shown as mean ± s.d. or photographs from one representative of three independent experiments. *P < 0.05, **P < 0.01, ^▲^*P* > 0.05

Although the DEN-induced hepatocarcinogenesis model has been widely used for its ease and consistency in generating HCC as well as HcPCs, a recent study determined that tumors of Stelic Animal Model (STAM), by using streptozotocin (STZ) and high-fat diet (HFD) to mimic NASH-induced HCC, were the most molecularly similar to human HCC among the available hepatocarcinogenesis mouse models [23]. We thus generated STAM tumors in *Rig-I*^*f/f*^ and *Rig-I*^*hep−/−*^ mice. Interestingly, hepatocyte-specific RIG-I deficiency nearly abolished NASH-induced hepatocarcinogenesis, suggesting the tumor-promotive role of RIG-I in NASH-induced HCC, which is opposite to the tumor-suppressive role of RIG-I in the DEN model (Fig. 5a, b, Additional file 2: Fig. S5a, b). In another NASH-induced HCC mouse model that western diet (WD) plus CCl_4_ injection, hepatic RIG-I deficiency also markedly inhibited hepatocarcinogenesis (Additional file 2: Fig. S5c). The generated STAM tumors of *Rig-I*^*f/f*^ and *Rig-I*^*hep−/−*^ mice were then analyzed. Although the pathological features of tumors were similar, we interestingly found that the NASH features in nontumor liver tissues, including ballooning and inflammation, were induced in *Rig-I*^*f/f*^ mice, while abolished by hepatic RIG-I deficiency (Fig. 5c). Moreover, hepatic oil red O staining also determined that lipid accumulation was abolished by RIG-I deficiency in STAM model (Fig. 5d). Thus, these data indicate that hepatic RIG-I deficiency may suppress NASH progression, and then NASH-induced hepatocarcinogenesis.

The methionine and choline deficient diet (MCD) and choline deficient-high-fat diet (CD-HFD) were then used to induce NASH in *Rig-I*^*f/f*^ and *Rig-I*^*hep−/−*^ mice to confirm the inhibited NASH progression by hepatic RIG-I deficiency. We found that NASH progression, the induced hepatic proinflammatory IL-6, and the fibrosis feature were all markedly suppressed in *Rig-I*^*hep−/−*^ livers of MCD model (Fig. 5e, Additional file 2: Fig. S5d, e, f), and NASH features were also abolished in *Rig-I*^*hep−/−*^ mice of CD-HFD model (Additional file 2: Fig. S5g). Furthermore, hepatic oil red O staining confirmed that lipid accumulation was inhibited by RIG-I deficiency in these NASH models (Fig. 5f, Additional file 2: Fig. S5h). Hence, we conclude that hepatic RIG-I deficiency suppresses NASH and NASH-induced HCC, and the tumor-promotive role of RIG-I in NASH-induced HCC is opposite to the tumor-suppressive role of RIG-I in DEN-induced hepatocarcinogenesis.

### Hepatic RIG-I deficiency suppressed the occurrence of steatosis, especially by the diminished hepatic cholesterol accumulation

As NASH-induced HCC goes through steatosis, NASH, fibrosis, cirrhosis, and HCC, we next examined whether hepatic RIG-I deficiency could inhibit the development of the first step steatosis. Using HFD to induce simple steatosis but minor NASH, we found that hepatic RIG-I deficiency suppressed HFD-induced steatosis as compared to that in *Rig-I*^*f/f*^ mice (Fig. 5g, Additional file 2: Fig. S5i). Hepatic oil red O staining also determined that lipid accumulation was abolished by RIG-I deficiency in the HFD model (Fig. 5h). The increase in body and liver weight induced by HFD was also suppressed in *Rig-I*^*hep−/−*^ mice (Additional file 2: Fig. S5j). Thus, hepatic RIG-I deficiency inhibits the occurrence of the first step steatosis, which then abolishes the following NASH and NASH-induced HCC. Moreover, we examined RIG-I expression in the liver of HFD-induced steatosis, and found the increased RIG-I in the steatosis liver (Additional file 2: Fig. S5k). Together, RIG-I expression is increased by hepatic steatosis, and RIG-I deficiency in hepatocytes abolishes steatosis development.

The accumulation of triglyceride (TG) and total cholesterol (TC) in hepatocytes is the main feature of hepatic steatosis, and they were then examined. In HFD-treated mice, both hepatic TG and TC were markedly suppressed in *Rig-I*^*hep−/−*^ mice (Fig. 5i). Remarkably, hepatic TC was equivalent between ND and HFD groups of *Rig-I*^*hep−/−*^ mice, suggesting the most markedly suppressed hepatic TC by RIG-I deficiency, and HFD could not increase hepatic cholesterol in *Rig-I*^*hep−/−*^ mice (Fig. 5i). Furthermore, the increased serum cholesterol in HFD-treated mice was abolished by hepatic RIG-I deficiency, while serum TG was not significantly influenced (Fig. 5j). Hence, RIG-I deficiency in hepatocytes abolished the HFD-induced increase in both hepatic and serum cholesterol, which may be responsible for the abolished steatosis in *Rig-I*^*hep−/−*^ mice. As atherosclerosis (AS) is the most threatening extrahepatic disease mediated by the increased serum cholesterol during hepatic steatosis, we also examined whether RIG-I deficiency could inhibit AS progression. Using the AS model of HFD-treated *ApoE* knockout mouse, we found that RIG-I deficiency markedly suppressed AS progression (Additional file 2: Fig. S5l, m). Thus, we conclude that RIG-I deficiency abolishes hepatic cholesterol accumulation to suppress both hepatic steatosis and high serum cholesterol-mediated extrahepatic disease.

As RIG-I expression is increased in the steatosis liver, we then examined its expression in NASH livers, and found that RIG-I expression was decreased in both NASH livers and NASH-induced HCC tissues (Additional file 2: Fig. S5n). Considering liver injury and inflammation are the typical features of NASH as compared to hepatic simple steatosis, and IL-6 is determined to decrease hepatic RIG-I expression (Fig. 1c, d), we also found that RIG-I decrease in NASH was suppressed by IL-6 knockout or hepatic IL-6 receptor knockout, thus suggesting that proinflammatory cytokine IL-6 mediates RIG-I decrease in NASH (Additional file 2: Fig. S5o). Together, we conclude that although hepatic RIG-I expression is increased in the first step steatosis stage to promote lipid accumulation and steatosis development, its expression is decreased in the following NASH and NASH-induced HCC, which is mediated by the proinflammatory cytokine IL-6.

### Methylated RIG-I associates AMPKα to inhibit HMGCR phosphorylation, thus increasing HMGCR enzymatic activity and enhancing cholesterol synthesis

**Fig. 6:**
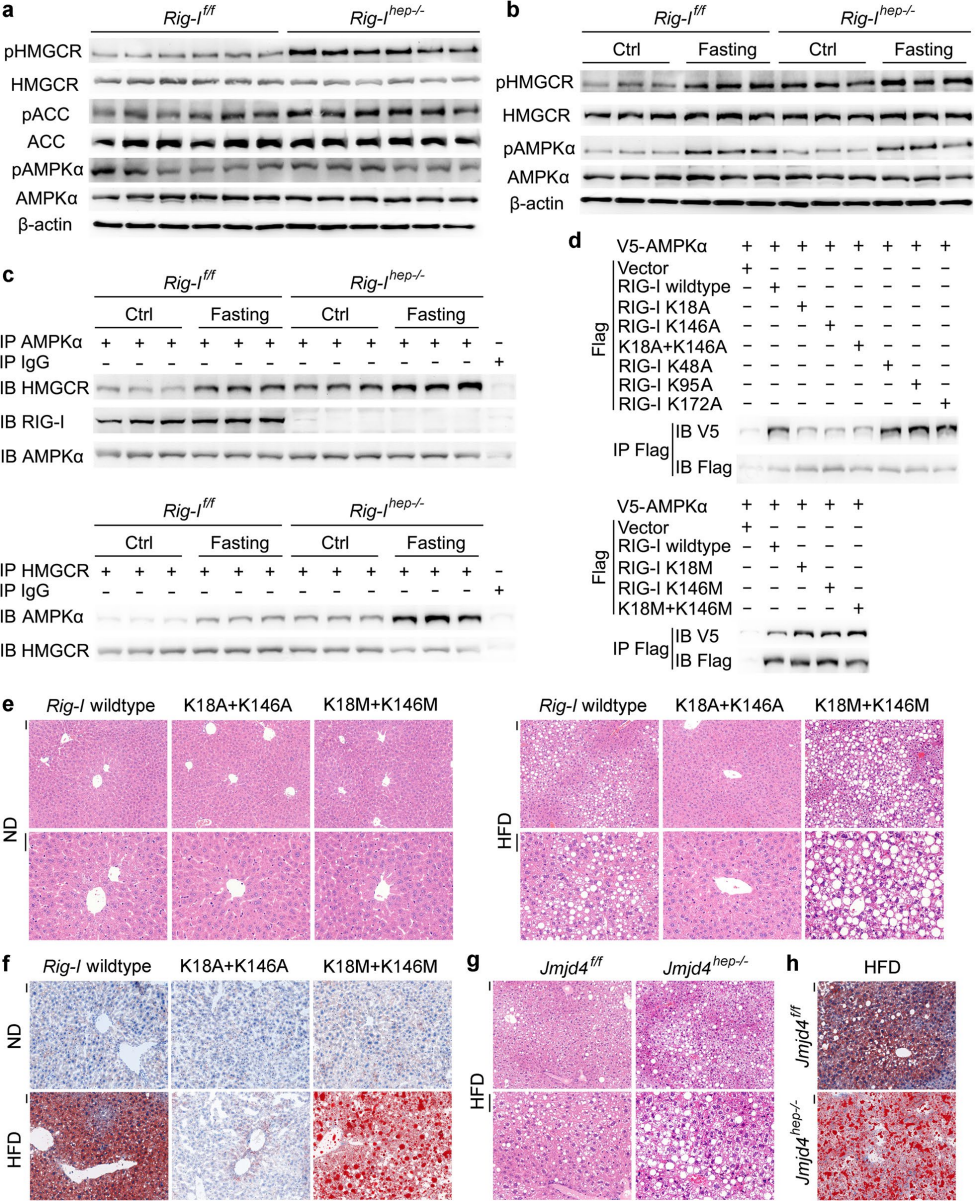
Methylated RIG-I associates AMPKα to inhibit HMGCR phosphorylation and enhance cholesterol synthesis. **a** HMGCR, ACC, AMPKα, and their phosphorylation were examined in liver tissues of male *Rig-I*^*f/f*^ and *Rig-I*^*hep−/−*^ mice. **b** HMGCR, AMPKα, and their phosphorylation were examined in liver tissues of male *Rig-I*^*f/f*^ and *Rig-I*^*hep−/−*^ mice fasting for 16 h. **c** The AMPKα-HMGCR interaction and AMPKα-RIG-I interaction upon fasting for 16 h were evaluated using immunoprecipitation in liver tissues from male *Rig-I*^*f/f*^ and *Rig-I*^*hep−/−*^ mice. **d** V5-tagged AMPKα and Flag-tagged RIG-I mutants as indicated were transfected into HHL5 hepatocyte cell line, and their association was tested by immunoprecipitation. **e**, **f** HE (**e**) and oil red O (**f**) staining were analyzed in the liver tissues of HFD model in male wildtype, RIG-I K18M+K146M or K18A+K146A mutant mice. Scale bars: 20 μm. **g**, **h** HE (**g**) and oil red O (**h**) staining were analyzed in the liver tissues of HFD model in male *Jmjd4*^*f/f*^ and *Jmjd4*^*hep−/−*^ mice. Scale bars: 20 μm. Data are shown as photographs from one representative of three independent experiments

To elucidate the mechanism responsible for RIG-I deficiency-mediated inhibition of cholesterol accumulation and steatosis, we performed the transcriptome analysis between the livers of *Rig-I*^*f/f*^ and *Rig-I*^*hep−/−*^ mice. As the different hepatic lipid accumulation following HFD treatment in *Rig-I*^*f/f*^ and *Rig-I*^*hep−/−*^ mice may influence the expression of the metabolic enzymes, we chose the livers under normal diet for analysis. The mRNAs of genes involved in lipid uptake, synthesis, transport, and excretion were not significantly influenced by hepatic RIG-I deficiency (Additional file 2: Fig. S6a). We then presumed that the protein levels of the cholesterol metabolic genes and/or their phosphorylation might be modulated by hepatic RIG-I deficiency, and screened them using Western blot. Among them, the phosphorylation of HMGCR, the rate-limiting enzyme for cholesterol synthesis and its phosphorylation leads to inactivation [28], was the most markedly increased in *Rig-I*^*hep−/−*^ livers (Fig. 6a). Thus, the phosphorylation of HMGCR, leading to the inactivation of cholesterol synthesis, is increased by hepatic RIG-I deficiency, which may be responsible for the most significantly suppressed cholesterol accumulation and hepatic steatosis in *Rig-I*^*hep−/−*^ mice.

As HMGCR phosphorylation and inactivation are mediated by upstream activated kinase AMPKα [28], we next examined the association between AMPKα and HMGCR to analyze whether their association was modulated by RIG-I. In *RIG-I*^*hep−/−*^ livers, both the basal level of HMGCR phosphorylation and its induction by fasting or metformin treatment were significantly enhanced, while the phosphorylation of upstream AMPKα was not influenced (Fig. 6b, Additional file 2: Fig. S6b), suggesting that AMPKα activation is not influenced by RIG-I deficiency. RIG-I was found to constitutively associate with AMPKα, and both the basal level and induced association between AMPKα and downstream HMGCR were significantly enhanced by hepatic RIG-I deficiency (Fig. 6c, Additional file 2: Fig. S6c), determining that AMPKα-HMGCR interaction is inhibited by RIG-I-AMPKα association. Moreover, the N-terminal CARD domain of RIG-I associated with the kinase domain of AMPKα, which was also responsible for the association between AMPKα and C-terminal catalytic domain of HMGCR (Additional file 2: Fig. S6d, e). Together, we conclude that hepatic RIG-I constitutively associates with AMPKα to impede HMGCR, thus inhibiting HMGCR phosphorylation and enhancing its enzymatic activity to synthetize cholesterol.

Since PTM of proteins is important for protein–protein interaction, and methylation at K18, K146, and acetylation at K48, K95, K172 were found to be constitutively existed in RIG-I, we further examined whether these PTMs could influence RIG-I-AMPKα association. The respective mutants at these sites were used, and interestingly, only K18A and K146A mutants mimicking demethylation markedly inhibited RIG-I-AMPKα association, while K18M and K146M mutants mimicking mono-methylation enhanced their association, suggesting that the constitutive K18 and K146 methylation of RIG-I is responsible for its interaction with AMPKα (Fig. 6d). Together with the result that IL-6 induced the demethylation of RIG-I at these two sites, we confirmed that the constitutive RIG-I-AMPKα association was inhibited by IL-6 administration (Additional file 2: Fig. S6f). In conclusion, K18 and K146 methylated RIG-I constitutively associates with AMPKα to inhibit HMGCR phosphorylation, thus enhancing HMGCR enzymatic activity and cholesterol synthesis.

To confirm the enhanced cholesterol synthesis mediated by constitutive methylated RIG-I, we examined the HFD-induced hepatic steatosis in K18A+K146A mutant mice mimicking demethylated RIG-I, and in K18M+K146M mutant mice mimicking mono-methylated RIG-I. In contrast to those in wildtype mice, RIG-I K18A+K146A mice showed abolished hepatic steatosis and cholesterol accumulation following HFD treatment, while K18M + K146M promoted hepatic steatosis (Fig. 6e, f, Additional file 2: Fig. S6g). The STAM tumor models were also performed, and RIG-I K18A+K146A mice nearly abolished while K18M + K146M promoted STAM hepatocarcinogenesis (Additional file 2: Fig. S6h). Moreover, as JMJD4 is responsible for the demethylation of RIG-I at K18 and K146, we confirmed that HFD-induced steatosis and STAM hepatocarcinogenesis were both promoted in *Jmjd4*^*hep−/−*^ mice, which were similar to those in RIG-I K18M+K146M mutant mice (Fig. 6g, h, Additional file 2: Fig. S6i, j). Altogether, we conclude that hepatic steatosis is promoted by the constitutive methylated RIG-I at K18 and K146, while suppressed by JMJD4-mediated RIG-I demethylation.

### RIG-I expression and methylation may be correlated to human hepatocarcinogenesis, prognosis, and NAFLD progression

**Fig. 7:**
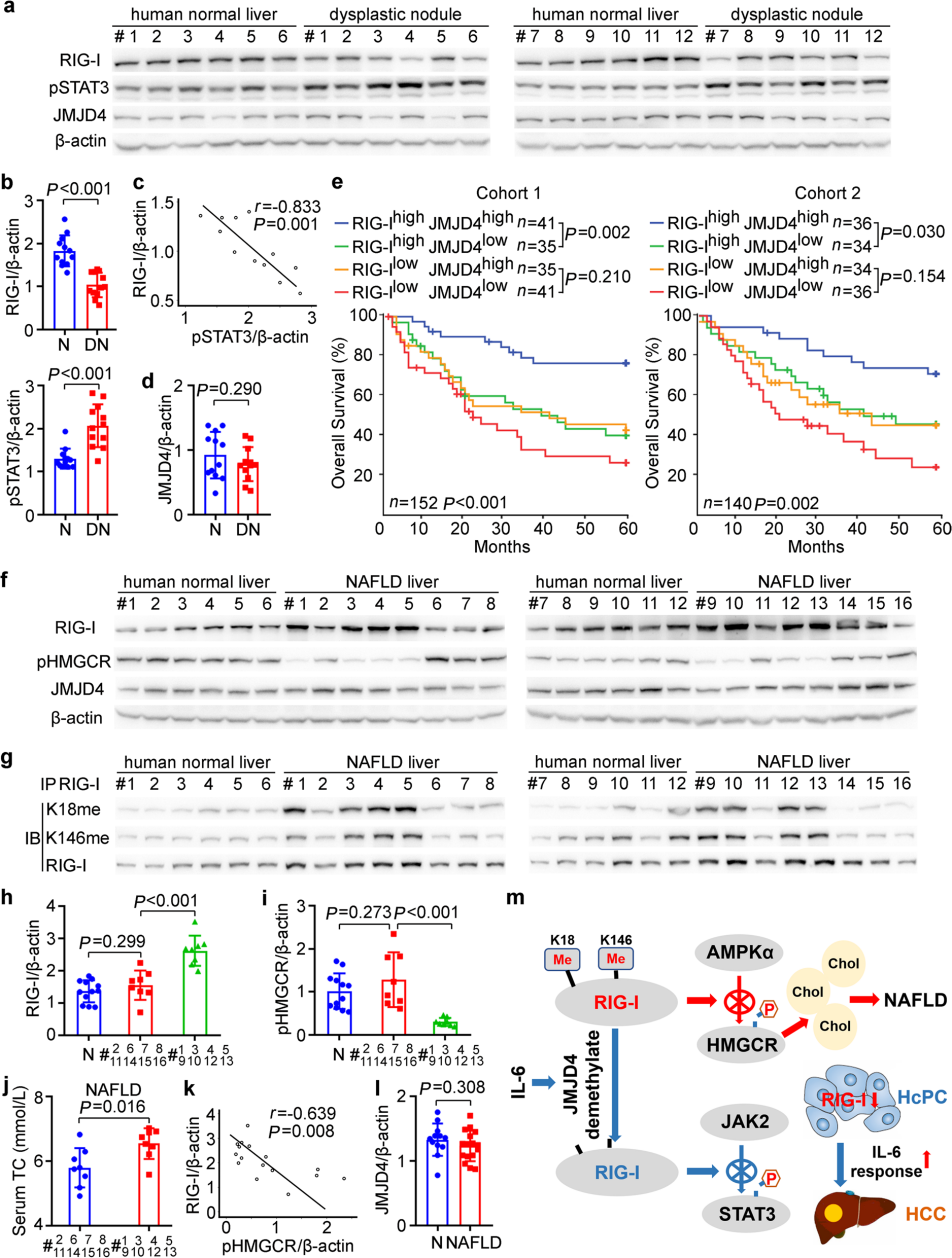
RIG-I expression and methylation may be correlated to human hepatocarcinogenesis, prognosis, and NAFLD progression. **a** RIG-I, JMJD4, and STAT3 phosphorylation were examined in human normal liver tissues and dysplastic nodule tissues from the indicated patients. **b** Quantified RIG-I expression and STAT3 phosphorylation in human normal liver (N) tissues and dysplastic nodule (DN) tissues were shown (*n* = 12, unpaired *t*-test). **c** The correlation between RIG-I expression and STAT3 phosphorylation in human dysplastic nodule tissues was analyzed by Pearson’s correlation coefficient assay. **d** Quantified JMJD4 protein level in human normal liver (N) tissues and dysplastic nodule (DN) tissues were shown (*n* = 12, unpaired *t*-test). **e** Kaplan–Meier survival curves of overall survival based on dichotomized *RIG-I* and *JMJD4* mRNA expression in HCC tissues of Cohort 1 and 2. The median levels of *RIG-I* and *JMJD4* expression in each cohort were used as the cutoff, with log-rank test for significance. **f** RIG-I, JMJD4, and HMGCR phosphorylation were examined in human normal liver tissues and NAFLD tissues from the indicated patients. **g** Methylated RIG-I at K18 or K146 were examined in the precipitates using RIG-I antibody from human normal liver tissues and NAFLD tissues as in **f**. **h**, **i** Quantified RIG-I protein level (**h**) and HMGCR phosphorylation level (**i**) in human normal liver tissues (N, *n* = 12) and the indicated NAFLD tissues (*n* = 8, unpaired *t*-test) were shown. **j** Serum TC of the indicated NAFLD patients were shown (*n* = 8, unpaired *t*-test). **k** The correlation between RIG-I expression and HMGCR phosphorylation in human NAFLD tissues was analyzed by Pearson’s correlation coefficient assay. **l** Quantified JMJD4 protein level in human normal liver (N, *n* = 12) tissues and NAFLD (*n* = 16, unpaired *t*-test) tissues were shown. **m** Working model for the mechanism of constitutive methylated RIG-I at K18 and K146 inhibits HMGCR phosphorylation to promote cholesterol synthesis and NAFLD progression, while IL-6-induced and JMJD4-mediated RIG-I demethylation inhibits STAT3 phosphorylation to suppress hepatocarcinogenesis, and decreased RIG-I in HcPCs enhances IL-6 effects and drives them to established HCC. Data are shown as mean ± s.d., survival curves, or photographs as indicated

In order to analyze the correlation of RIG-I expression to human hepatocarcinogenesis, we examined that in hepatic dysplastic nodules, which represent precancerous lesions of HCC. RIG-I expression was decreased while phosphorylated STAT3 was increased in human dysplastic nodules (Fig. 7a, b), which is consistent with those determined in mouse HcPCs. RIG-I expression in dysplastic nodules was significantly reverse-correlated to STAT3 phosphorylation, implicating that decreased RIG-I may contribute to STAT3 activation in human dysplastic nodules (Fig. 7c). Additionally, hepatic JMJD4 expression differed between individuals, and was not statistically changed between normal livers and dysplastic nodules (Fig. 7d). Thus, decreased RIG-I in hepatic dysplastic nodules may be correlated to human hepatocarcinogenesis.

We next analyzed the correlation of RIG-I and JMJD4 expression in HCC tissues to the prognosis of patients. Using Cohort 1 (*n* = 152) and Cohort 2 (*n* = 140) HCC patients as we described previously [13], we determined that high RIG-I in HCC predicts better disease-free survival and overall survival (Additional file 2: Fig. S7a, b). Moreover, the group of patients with high RIG-I and high JMJD4 in HCC had the significant best outcome (Fig. 7e, Additional file 2: Fig. S7c). The patients with high RIG-I and low JMJD4 in HCC had significant worse outcome than that of the high RIG-I and high JMJD4 group (Fig. 7e, Additional file 2: Fig. S7c), suggesting that RIG-I and JMJD4 co-operatively predict better prognosis, and JMJD4-mediated RIG-I demethylation may be important in the identification of HCC prognosis.

To examine the correlation of RIG-I expression and methylation to human NAFLD, we analyzed RIG-I in the liver tissues of NAFLD patients. RIG-I expression was significantly increased in the livers from at least 8 of 16 (50%) NAFLD patients, and the level of methylated RIG-I at K18 and K146 were also significantly increased in these 8 NAFLD livers (Fig. 7f–h, Additional file 2: Fig. S7d, e). Although HMGCR phosphorylation differed between individuals and was not significantly changed in NAFLD livers (Additional file 2: Fig. S7d), HMGCR phosphorylation was significantly lower while serum cholesterol was significantly higher in the 8 NAFLD livers with increased RIG-I than in those without significantly increased RIG-I (Fig. 7i, j). Furthermore, RIG-I expression in NAFLD livers was significantly reverse-correlated to HMGCR phosphorylation (Fig. 7k), which is consistent with the RIG-I-mediated inhibition of HMGCR phosphorylation determined in mouse livers. Additionally, hepatic JMJD4 expression also differed between individuals and was not statistically changed in human NAFLD livers (Fig. 7l). Thus, we conclude that increased constitutive methylated RIG-I at K18 and K146 in NAFLD liver may be correlated to human NAFLD progression.

As RIG-I expression is determined to be increased in the liver steatosis stage while decreased in the following NASH and NASH-induced HCC in mouse models, we then examined RIG-I expression in human NASH and NASH-HCC tissues. In the available liver tissues from NASH patients and HCC tissues from NASH-HCC patients, RIG-I expression was found to be significantly lower while STAT3 phosphorylation was significantly higher than those in human normal liver tissues (Additional file 2: Fig. S7f–i), which is consistent with the decreased RIG-I and increased STAT3 activation determined in mouse NASH and NASH-induced HCC (Additional file 2: Fig. S5n). Together, we conclude that although hepatic RIG-I expression is increased in the first step steatosis stage to promote cholesterol synthesis and lipid accumulation, its expression is decreased in the following NASH and NASH-induced HCC, which may contribute to the inflammation-driven hepatocarcinogenesis.

## Discussion

Here, we interestingly find that hepatocarcinogenesis phenotypes modulated by hepatocyte-specific RIG-I deficiency are inverse in two commonly used mouse models, which are RIG-I promotes STAM HCC representing NASH-induced carcinogenesis whereas suppresses DEN-induced inflammation and carcinogenesis. Mechanistically, constitutive methylated RIG-I at K18 and K146 enhances hepatic cholesterol synthesis and lipid accumulation, thus promoting steatosis and the following NASH and NASH-HCC, whereas JMJD4-demethylated RIG-I suppresses IL-6 response and inflammation-induced hepatocarcinogenesis. More interestingly, JMJD4-demethylated RIG-I is beneficial in preventing both steatosis and inflammation-induced carcinogenesis, while the constitutive methylated RIG-I tends to promote them both, which are determined in RIG-I K18A+K146A mice and K18M+K146M mice in vivo. Thus, mutations at these two lysine sites of *RIG-I* gene may be correlated to human hepatic steatosis and carcinogenesis. Based on the public large sequencing data, we find that an SNP site exists at K18 (rs763359633, C > G, Lys > Asn), while no SNP is suggested at K146. However, it remains unknown whether this SNP site is correlated to human liver diseases, which is worth further analyzing intensively.

As an important PTM manner, protein methylation has been linked to transcriptional regulation in histones, and to protein–protein interaction between non-histones [17, 29]. Although a set of PTM of RIG-I have been identified, including phosphorylation, ubiquitination, and acetylation [16], whether RIG-I is methylated and the potential role of methylation in regulating RIG-I function are still unknown currently. Here, the constitutive K18 and K146 methylation of RIG-I and IL-6-induced demethylation by demethylase JMJD4 are determined, which is responsible for the IL-6-induced RIG-I-STAT3 association. However, it is still unknown that the underlying mechanism and corresponding methylase for the constitutive K18 and K146 methylation in RIG-I, which may maintain the cellular response to IL-6 in the quiescent condition and the activity for cholesterol synthesis. Whether K18 and K146 of RIG-I return to be methylated when IL-6 is withdrawn, and which methyltransferase mediates this process remain to be investigated. Moreover, as demethylated RIG-I not only suppresses inflammation-induced hepatocarcinogenesis, but also inhibits hepatic steatosis and the following NASH and NASH-HCC, and considering that inhibition of protein lysine methylation has been suggested to be potential therapeutic target and that clinical trials of several inhibitors have shown promising results [30, 31], we presume that targeting the methylation of RIG-I may bear considerable therapeutic potential for the intervention of both NAFLD and HCC, although further structural data of RIG-I methylation remain to be investigated for the design of specific inhibitors.

During inflammation-induced hepatocarcinogenesis, we show that IL-6 induces RIG-I expression and RIG-I-STAT3 association to feedback suppress IL-6-STAT3 signaling in the early phase, while decreases RIG-I expression in HcPCs to enhance IL-6 response and hepatocarcinogenesis in the late phase. In this process, initiated hepatocytes acquire the ability to autocrine IL-6 and drive their malignant progression, thus becoming HcPCs, whose phenotype is CD44 positive also induced by IL-6-STAT3 signaling [4, 32]. Here, not only IL-6 production but also IL-6 response are determined to be enhanced in HcPCs, and increased IL-6 response is mediated by the decreased RIG-I, which is also induced by IL-6 in the late phase, thus suggesting the feedforward vicious autocrined IL-6 and increased IL-6 response in the driving of hepatocarcinogenesis, especially in the stage from HcPCs to established HCC. Moreover, as IL-6 plays critical roles in the carcinogenesis of series types of cancer, such as hematologic tumors, gastrointestinal cancers, and urogenital neoplasms, whether decreased RIG-I-mediated vicious augment of IL-6 response is a common mechanism for IL-6-promoted malignancies may raise interesting future work in this field. Additionally, although RIG-I is determined to be increased upon steatosis while decreased during NASH, its roles in NASH progression remain not investigated in this study. A recent report also found the decreased RIG-I in the NASH liver, which leads to impaired autophagy and cell death [33]. The potential roles of RIG-I especially its methylation in the NASH stage still need to be further investigated in the future.

RIG-I is a well-established intracellular sensor for host recognition of invading RNA virus in the innate immune response, and is also important for the differentiation and activation of T cells in adaptive immune system [11, 34]. However, the roles of RIG-I methylation or demethylation at K18 and K146 in innate immune sensing and activation of immune response are still unknown, which may also be interesting and attractive. Moreover, RIG-I is determined to be expressed in the parenchymal cells of organs, such as in hepatocytes in this study, which may suggest its specific roles associating with the functions of the organs. Other immune sensors or immune molecules may be also expressed in the parenchyma of organs, and their functions may be not restricted in the immune activation or regulation. Currently, as molecules or factors of other systems such as metabolism have been intensively investigated in the regulation of immune response, the immune molecules are probably to be also important in the regulation of other systems and progression of other diseases. For example, a current work reported that stress-induced IL-6 from brown adipocytes could enhance hepatic gluconeogenesis and hyperglycemia [35], which may be in line with our data that IL-6-induced demethylated RIG-I disassociates with AMPKα and may enhance HMGCR phosphorylation to suppress cholesterol synthesis. The potential interaction or cross talk between immune system and other systems especially metabolism at the level of intracellular molecules are promising topics in future studies.

RIG-I, also known as DDX58, belongs to the DEAD/DEAH box helicase (DDX) protein family. DDX family is the largest family of RNA helicases and contains vital players of RNA metabolism. Members of this family share conserved helicase domain for RNA binding and unwinding properties, possessing roles in RNA transcription, splicing, modification, exporting, translation, and degradation [36]. Other than helicase domain, DDX family members contain their specific domains for the interaction between proteins. For RIG-I, the N-terminal CARD domain mediates its interaction with downstream MAVS upon viral infection, and with STATs upon cytokine stimulation determined here and in our previous study [13]. Thus, the functions of DDX family members are not restricted in RNA binding, other domains contained in individual members may expand their roles in diverse properties. Additionally, as the helicase domain of RIG-I or other DDX members can constitutively associate with intracellular RNAs [37], the helicase domain binding host RNAs may also modulate their protein–protein interaction, which needs further investigation.

## Conclusions

In summary, we propose that hepatic RIG-I is constitutively mono-methylated at K18 and K146, and increased methylated RIG-I during steatosis promotes cholesterol synthesis by inhibiting HMGCR phosphorylation, thus promoting NAFLD progression. Upon hepatic inflammation, inflammatory circumstances such as IL-6 promote JMJD4-mediated RIG-I demethylation at K18 and K146, thus restricting STAT3 activation and oncogenic transformation, and decreased RIG-I expression in liver cancer progenitor HcPCs enhances IL-6 effects and drives them to fully established HCC (Fig. 7m). Together, we find that JMJD4-demethylated RIG-I at K18 and K146 is an essential mechanism to inhibit hepatic steatosis and carcinogenesis.

## Supplementary Information

**Additional file 1.** Supplementary Methods.

**Additional file 2.** Supplementary Figures.

## Data Availability

The accession number of the RNA-seq data is SRA: PRJNA664713. All the unprocessed gels and images, and the original source data for all figures are available at Mendeley Data Reserved https://data.mendeley.com/datasets/jvn3t64p8j/6.

## Abbreviations

AMPKα: AMP-activated protein kinase α
AS: Atherosclerosis
CARD: Caspase-recruiting domain
CD-HFD: Choline deficient-high-fat diet
DAMP: Damage-associated molecular pattern
DDX: DEAD/DEAH box helicase
DEN: Diethylnitrosamine
DKO: Double knockout
HCC: Hepatocellular carcinoma
HcPC: Hepatocellular carcinoma progenitor cell
HFD: High-fat diet
HMGCR: 3-Hydroxy-3-methylglutaryl-CoA reductase
IFN: Interferon
IL-6: Interleukin-6
JAK2: Janus kinase 2
JMJD4: Jumonji C domain-containing demethylase 4
MCD: Methionine and choline deficient diet
MS: Mass spectrometry
NAFLD: Nonalcoholic fatty liver disease
NASH: Nonalcoholic steatohepatitis
ND: Normal diet
PTM: Post-translational modifications
qRT-PCR: Quantitative RT-PCR
RIG-I: Retinoic acid-inducible gene-I
SREBP: Sterol-regulatory element binding protein
STAM: Stelic animal model
STAT3: Signal transducer and activator of transcription 3
STZ: Streptozotocin
TC: Total cholesterol
TG: Triglyceride
TLR: Toll-like receptor
WD: Western diet
