# Supplementary material for: JMJD4-demethylated RIG-I prevents hepatic steatosis and carcinogenesis
Source: J Hematol Oncol. 2022 Nov 4;15:161. doi: 10.1186/s13045-022-01381-6 (PMC9636772; doi:10.1186/s13045-022-01381-6)
Supplement: Supplementary file 1 — Additional file 1. Supplementary Methods. [file 13045_2022_1381_MOESM1_ESM.docx]

**Additional file 1: Supplementary Methods**

**Reagents**

Antibodies specific to RIG-I (3743), pSTAT3 Y705 (4113), STAT3 (9139), JAK1 (3344), pJAK2 (3771), JAK2 (3230), SHP1 (3759), SHP2 (3397), SOCS1 (3950), SOCS3 (52113), PIAS1 (3550), PIAS3 (4164), pACC (11818), ACC (3676), pAMPKα (50081), AMPKα (5832), cleaved-caspase 3 (9661), pH2AX (9718), and horseradish peroxidase‑coupled secondary antibodies (7074 and 7076) were from Cell Signaling Technology (Danvers, MA). Antibodies specific to Flag-tag (F1804), β-actin (A5441), and HMGCR (SAB4200529) were from Sigma-Aldrich (St. Louis, MO). Antibodies specific to RIG‑I (ab45428), Ki67 (ab16667), Ly6G (ab238132), and V5-tag (ab9116) were from Abcam (Cambridge, MA). Antibody specific to pHMGCR (orb251472) was from Biorbyt (United Kingdom). Antibody specific to JMJD4 was constructed and purified by Proteintech Group (Wuhan, China). Antibodies specific to mono-methylated RIG-I at K18 or K146 were developed using the indicated synthesized antigenic peptides, and rabbit polyclonal antibodies to these peptides were constructed and purified by Abmart (Shanghai, China) as we described previously [1, 2]. The antibody specificity was validated by dot blot analysis. Antibody specific to CD44-PE (130-102-606) and Anti-PE MicroBeads (130-048-801) were from Miltenyi Biotec (Germany). Protein G Agarose (P4691), Anti-Flag M2 Affinity Gel (A2220), and DEN (N0258) were from Sigma‑Aldrich. Recombinant mouse IL-6 (575706) was from BioLegend (San Diego, CA). Fetal Bovine Serum (FBS, 10099141C), DMEM (11965092), and RPMI 1640 (11875093) were from Gibco (Shanghai, China).

**Cell lines and transfection**

The human hepatocyte cell line HHL5 was obtained from the Type Culture Collection of the Chinese Academy of Sciences (Shanghai, China), and cultured using RPMI 1640 with 10% FBS as routine. Cells were seeded and then transfected with plasmids using jetPRIME transfection reagent (114-15, Polyplus-transfection, France) according to the manufacturer’s protocol as we described previously [3].

**Isolation of primary hepatocytes and HcPCs**

Primary hepatocytes from eight-week-old male mice were isolated using two-step liver perfusion method including perfusion and digestion as described previously [4]. Primary hepatocytes were separated by centrifugation at 50 g for 5 minutes and purified on 50% Percoll solution (P4937, Sigma-Aldrich), and then resuspended in DMEM supplemented with 10% FBS and seeded into six-well plates at 1×10^7^ cells per well. For isolating HcPCs, livers of male mice five months post the initial DEN injection at postnatal day 15 were perfused, and nonaggregate and aggregate cells were isolated by filtration through 70 and 40 μm sieves. After dispersing the aggregates into single cells, HcPCs were enriched for CD44^+^ cells using magnetic beads as reported [4]. 1×10^3^ HcPCs were transplanted through intrasplenic injection to the male mice undergone Retrorsine (PHL84274, Sigma-Aldrich) injection before and CCl_4_ injection after transplantation as reported [4]. Tumor multiplicity and size were evaluated five months post transplantation.

**Molecular cloning of genes**

The genes and their various truncates in this study were amplified by PCR from the cDNA of human liver tissue, and subsequently cloned into pcDNA vectors with Flag or V5 tag. Each construct was confirmed by sequencing. Genes with site mutations were constructed by Fast Mutagenesis System (FM111-02, TransGen Biotech, Beijing, China) and confirmed by sequencing. The rAAV (serotype 8) vector expressing RIG-I under promoter CAG was constructed as we previously described [5, 6]. For AAV8 administration, 1×10^12^ vg AVV8 was injected through tail vein two weeks before the sacrifice.

**MS analysis**

RIG-I and its associated proteins were immunoprecipitated from HHL5 hepatocyte cell line treated with IL-6 (100 ng/ml) for 30 minutes, and the precipitates were washed, boiled, and loaded to SDS-PAGE. After Coomassie Blue staining, the RIG-I specific bands and the selected bands were cut and then analyzed in reverse-phase nanospray liquid chromatography-tandem mass spectrometry. The MS and spectra analysis were performed by PTM BIO (Hangzhou, China) as we described previously [1, 2, 7].

**RNA-seq**

Total RNA was extracted using TRIzol reagent (Invitrogen, Carlsbad, CA) and subjected to high throughput sequencing as we previously described [8]. Genes with FPKM values above 5.0 were used for subsequent analysis. The mRNA levels of genes associated with lipid uptake, synthesis, transport, and excretion were gathered and analyzed by Heml 1.0 software.

**RNA extraction and real-time PCR**

Total RNA was extracted from frozen HCC tissues, liver tissues, and primary cells using TRIzol reagent following the manufacturer's instructions. Real-time quantitative RT-PCR (qRT-PCR) analysis was performed using LightCycler (Roche, Switzerland) and SYBR RT-PCR kit (RR430B, Takara, Dalian, China) as previously described [4]. The qPCR primers for gene expression analysis were human *RIG-I* (forward: 5’-TGT GCT CCT ACA GGT TGT GGA-3’, reverse: 5’-CAC TGG GAT CTG ATT CGC AAA A-3’); human *JMJD4* (forward: 5’-CTG CTA CGG ACC TAC GGA GAC-3’, reverse: 5’-GCC CGC CTG TAT GTA CTC TTT C-3’); internal control human *β-actin* (forward: 5’-ACA ATG AGC TGC TGG TGG CT-3’, reverse: 5’-GAT GGG CAC AGT GTG GGT GA-3’); mouse *Rig-I* (forward: 5’-AGA GTG TCA GAA TCT CAG TCA-3’, reverse: 5’-CTG CTG CTC ATA GAC AGG AA-3’); mouse *Saa1* (forward: 5’-AGG GTT TTT TTC ATT TGT TC-3’, reverse: 5’-TCT GAG TTT TTC CAG TTA GC-3’); mouse *IL-6* (forward: 5’-TGA TGC ACT TGC AGA AAA CA-3’, reverse: 5’-ACC AGA GGA AAT TTT CAA TAG GC-3’); mouse *TNF-α* (forward: 5’-CAG GCG GTG CCT ATG TCT C-3’, reverse: 5’-CGA TCA CCC CGA AGT TCA GTA G-3’); internal control mouse *β-actin* (forward: 5’-AGT GTG ACG TTG ACA TCC GT-3’, reverse: 5’-GCA GCT CAG TAA CAG TCC GC-3’). The relative expression of the individual genes was normalized to that of internal control using 2^‑ΔΔCt^ cycle threshold method in each sample [9].

**Primer sequences for mice identification**

*Rig-I^f/f^* forward: 5’-AGG ATT GCT CTT GGT CAG-3’, reverse: 5’-CTG TGA AGT ATA GTT GAG TAG G-3’; *Rig-I* K18A+K146A WT forward: 5’-TGA GGC AGT TTC GAT TTC CTA TGG-3’, mutant forward: 5’-CTG CTG TCC ATT CCT TAT TCC ATA‑3’, common reverse: 5’-GTG GAA ACA CCC AGT GCA ATT TAC-3’; *Rig-I* K18M+K146M WT forward: 5’-TGA GGC AGT TTC GAT TTC CTA TGG-3’, WT reverse: 5’-GCT GTC TAC TCT AAT ATG GCC TGG-3’, mutant forward: 5’-GGC AGT TTC GAT TTC CTA TGG C-3’, mutant reverse: 5’-AGT GTC TCG GAT CTG TCT GAT TTC-3’; *Jmjd4^f/f^* forward: 5’-GCA GAG GAA GCC ATA CCT GAG AA-3’, reverse: 5’-ATC AAT GGG CCT CCA AAT GAT GG-3’; *IL-6^-/^*^-^ common forward: 5’‑TTC CAT CCA GTT GCC TTC TTG-3’, WT reverse: 5’‑TTC TCA TTT CCA CGA TTT CCC AG-3’, mutant reverse: 5’-CCG GAG AAC CTG CGT GCA ATC C-3’; *IL‑6ra^f/f^* forward: 5’-GAA GGA GGA GCT TGA CCT TGG-3’, reverse: 5’-AAC CAT GCC TAT CAT CCT TTG G-3’; Alb-Cre WT forward: 5’-TGC AAA CAT CAC ATG CAC AC-3’, mutant forward: 5’-GAA GCA GAA GCT TAG GAA GAT GG-3’, common reverse: 5’-TTG GCC CCT TAC CAT AAC TG-3’.

**Liver function and lipid test**

Liver function and serum lipid were evaluated by determining the activities of ALT and AST in the serum and serum lipid using an automatic biochemical analyzer FDC-7000i (Shanghai, China) according to the manufacturer’s instructions. Hepatic TC content was examined using Cholesterol Assay Kit (ab65390, Abcam), and hepatic TG content was examined using Triglyceride Determination Kit (TR0100, Sigma-Aldrich) according to the respective standard protocols.

**Histological analysis**

Paraffin-embedded tissue sections were stained with HE to visualize liver pathology, and were immunostained for the examination of pH2AX, cleaved-caspase 3, Ly6G, Ki67, and TUNEL staining as we described previously [5, 10]. Oil red O staining of frozen liver sections was performed to assess lipid droplet accumulation. Images were acquired and scanned with a light microscope (Pannoramic DESK, 3D HISTECH, Hungary).

**Immunoprecipitation and Western blot**

Cells or tissues were harvested and lysed with cell lysis buffer (9803, Cell Signaling Technology) supplemented with protease inhibitor cocktail (539134, Millipore). Protein concentrations of the lysates were measured with bicinchoninic acid (BCA) protein assay kit (ab102536, Abcam) and equalized with the lysis buffer. Equal amount of the extracts was used for immunoprecipitation, or loaded and subjected to SDS‑PAGE, transferred onto nitrocellulose membranes, and then blotted as we described previously [11]. β-actin was used as a loading control. Protein levels were quantified using Image J software and normalized to the internal control β-actin.

**References**

1. Chen K, et al. Methyltransferase SETD2-mediated methylation of STAT1 is critical for interferon antiviral activity. Cell. 2017;170:492-506.
2. Wang L, Wen M, Cao X. Nuclear hnRNPA2B1 initiates and amplifies the innate immune response to DNA viruses. Science. 2019;365:eaav0758.
3. Zheng Q, et al. Siglec1 suppresses antiviral innate immune response by inducing TBK1 degradation via the ubiquitin ligase TRIM27. Cell Res. 2015;25:1121-36.
4. Zhou Y, et al. Malignant progression of liver cancer progenitors requires lysine acetyltransferase 7-acetylated and cytoplasm-translocated G protein GαS. Hepatology. 2022 Mar 28. doi: 10.1002/hep.32487.
5. Han Y, et al. Tumor-induced generation of splenic erythroblast-like Ter-cells promotes tumor progression. Cell. 2018;173:634-48.
6. Han G, et al. Active tolerance induction and prevention of autoimmune diabetes by immunogene therapy using recombinant adenoassociated virus expressing glutamic acid decarboxylase 65 peptide GAD(500-585). J Immunol. 2005;174:4516-24.
7. Li X, et al. Methyltransferase Dnmt3a upregulates HDAC9 to deacetylate the kinase TBK1 for activation of antiviral innate immunity. Nat Immunol. 2016;17:806-15.
8. Zhang Q, et al. Tet2 is required to resolve inflammation by recruiting Hdac2 to specifically repress IL-6. Nature. 2015;525:389-93.
9. Livak KJ, Schmittgen TD. Analysis of relative gene expression data using real-time quantitative PCR and the 2^-ΔΔCt^ method. Methods. 2001;25:402-8.
10. Li Z, et al. microRNA-199a-3p inhibits hepatic apoptosis and hepatocarcinogenesis by targeting PDCD4. Oncogenesis. 2020;9:95.
11. Zheng Q, et al. Siglec1 suppresses antiviral innate immune response by inducing TBK1 degradation via the ubiquitin ligase TRIM27. Cell Res. 2015;25:1121-36.
