# Supplementary material for: JMJD4-demethylated RIG-I prevents hepatic steatosis and carcinogenesis
Source: J Hematol Oncol. 2022 Nov 4;15:161. doi: 10.1186/s13045-022-01381-6 (PMC9636772; doi:10.1186/s13045-022-01381-6)
Supplement: Supplementary file 2 — Additional file 2. Supplementary Figures. [file 13045_2022_1381_MOESM2_ESM.docx]

**Additional file 2: Supplementary Figures**

**
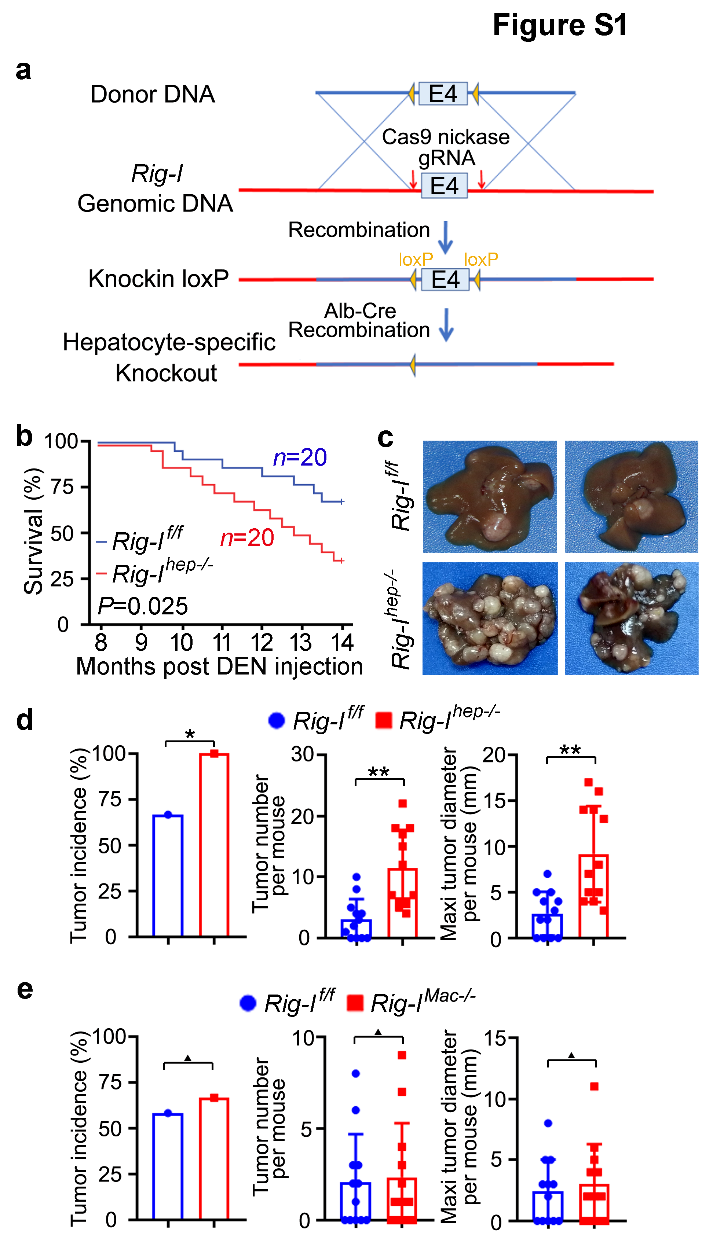
**

**Figure S1** IL-6-induced RIG-I decrease in HcPCs promotes hepatocarcinogenesis.

**a** Construction of *Rig‑I^hep‑/-^* mice.

**b** Kaplan-Meier survival curves of *Rig‑I^f/f^* and *Rig‑I^hep‑/-^* mice in the DEN-induced HCC mouse model (log-rank test, *n*=20).

**c** Representative livers of DEN plus CCl_4_‑induced HCC in male *Rig‑I^f/f^* and *Rig‑I^hep‑/-^* mice.

**d** Tumor incidence (chi-square test), number and maximum diameter (unpaired *t*-test) in **c** were analyzed (*n*=12).

**e** Tumor incidence (chi-square test), number and maximum diameter (unpaired *t*-test) of DEN-induced HCC in male *Rig-I^f/f^* and *Rig‑I^Mac‑/-^* mice were analyzed (*n*=12).

Data are shown as mean ± s.d. or photographs from one representative of three independent experiments. **P*<0.05, ***P*<0.01, ^▲^*P*>0.05.


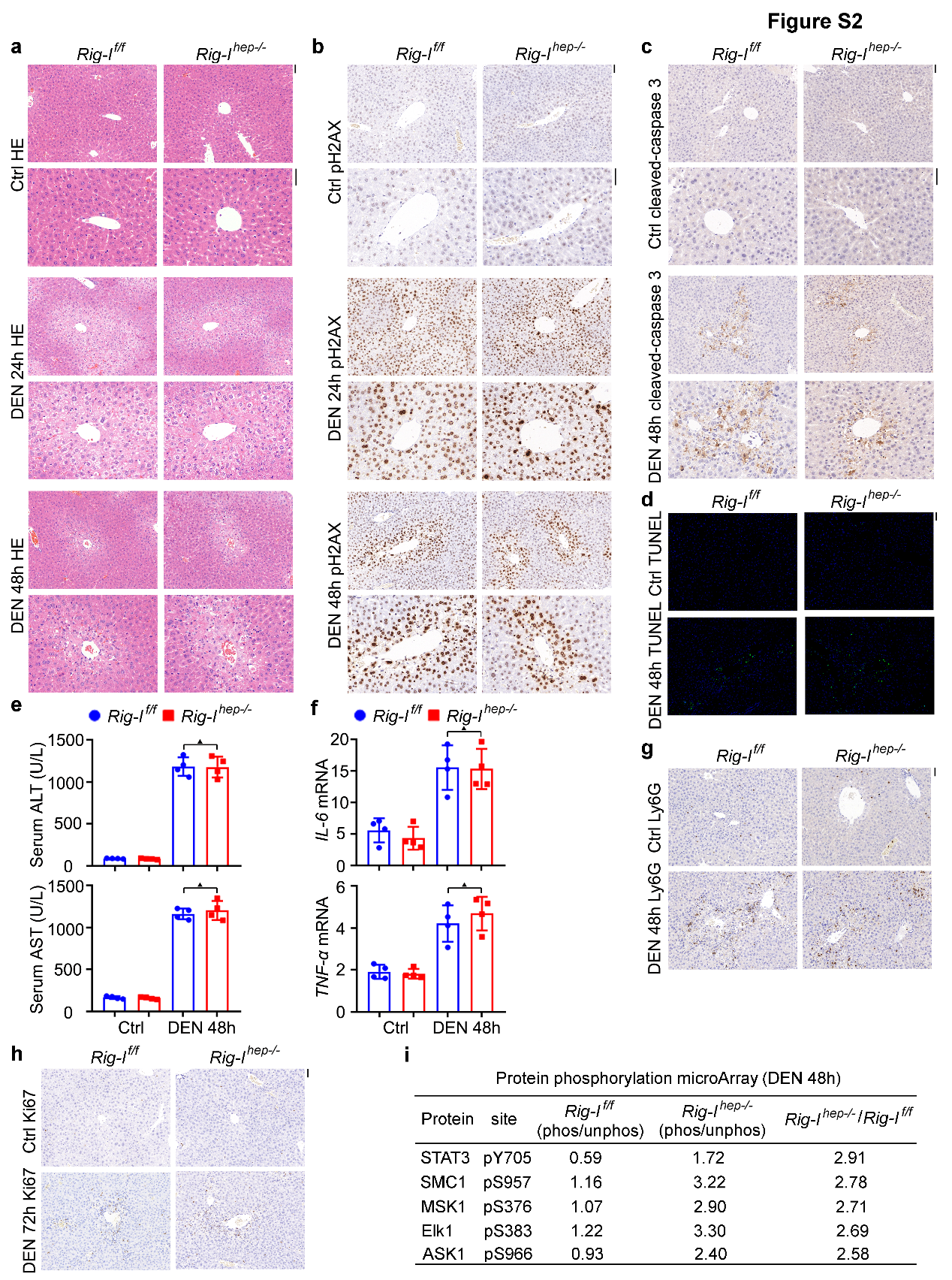


**Figure S2** Hepatocyte-specific RIG-I deficiency promotes DEN‑induced STAT3 phosphorylation in the liver.

**a-h** Eight-week-old male *Rig-I^f/f^* and *Rig‑I^hep‑/-^* mice were administrated with DEN for the indicated time periods, liver pathology was analyzed by HE staining (**a**), DNA damage was analyzed by pH2AX staining (**b**), hepatocyte apoptosis was analyzed by cleaved-caspase 3 (**c**) and TUNEL (**d**) staining, damage of liver function was analyzed by serum ALT and AST (**e**), *IL-6* and *TNF-α* mRNAs in liver tissues were analyzed by qRT-PCR (**f**), infiltration of leukocytes was analyzed by Ly6G staining (**g**), and compensatory hepatocyte proliferation was analyzed by Ki67 staining (**h**). Scale bars: 20 μm. For **e** and **f**, *n*=4, unpaired *t*-test.

**i** Eight-week-old male *Rig-I^f/f^* and *Rig‑I^hep‑/-^* mice were administrated with DEN for 48 hours, protein phosphorylation microArray of liver tissues was performed using PEX100 Phospho Explorer Array (Full Moon Biosystems). Top five most increased phosphorylation of intracellular signaling molecules in *Rig‑I^hep‑/-^* liver were shown as indicated. Phospho-specific antibody activities were normalized by the corresponding total protein (phos/unphos).

Data are shown as mean ± s.d. or photographs from one representative of three independent experiments. ^▲^*P*>0.05.

**
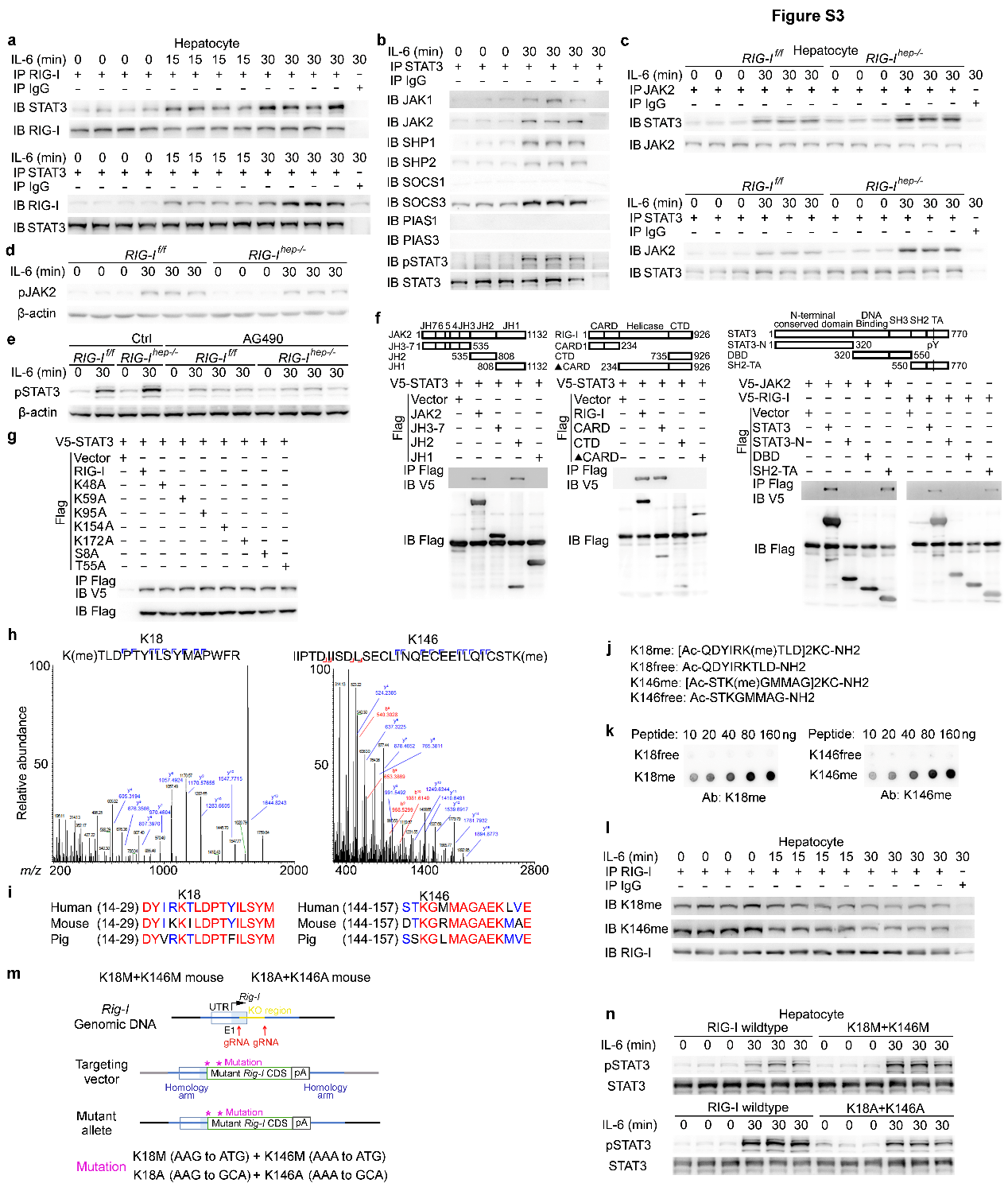
**

**Figure S3** IL-6 induces RIG-I demethylation to enhance RIG‑I‑STAT3 association and feedback impede JAK2-STAT3 interaction.

**a** RIG‑I‑STAT3 association induced by IL-6 was examined by immunoprecipitation in isolated male mouse primary hepatocytes.

**b** Interactions between STAT3 and JAK1, JAK2, SHP1, SHP2, SOCS1, SOCS3, PIAS1, or PIAS3 induced by IL-6 was examined by immunoprecipitation in male mouse liver tissues.

**c** IL-6-induced JAK2-STAT3 interaction was evaluated by immunoprecipitation in isolated primary hepatocytes from male *Rig-I^f/f^* and *Rig‑I^hep‑/-^* mice.

**d** IL-6-induced JAK2 phosphorylation was evaluated in liver tissues from male *Rig‑I^f/f^* and *Rig‑I^hep‑/-^* mice upon IL-6 stimulation.

**e** IL-6-induced STAT3 phosphorylation was evaluated in control or JAK2 inhibitor AG490-treated primary hepatocytes from male *Rig-I^f/f^* and *Rig‑I^hep‑/-^* mice as indicated.

**f** V5-tagged STAT3, Flag‑tagged JAK2 truncates and Flag-tagged RIG-I truncates as indicated were transfected, or Flag-tagged STAT3 truncates, V5‑tagged JAK2 and V5-tagged RIG-I as indicated were transfected into HHL5 hepatocyte cell line, and JAK2-STAT3 and RIG‑I‑STAT3 association were tested by immunoprecipitation.

**g** V5-tagged STAT3 and Flag-tagged RIG-I mutants as indicated were transfected into HHL5 hepatocyte cell line, and their association was tested by immunoprecipitation.

**h** Tandem mass spectrometry spectrum of RIG-I methylated K18 and K146 fragments. Detected productions are indicated in red (b ions) and blue (y ions).

**i** Sequence alignment of RIG-I K18 and K146 from the indicated species.

**j** Synthesized peptides used to generate antibodies specific to methylated RIG‑I at K18 or K146, and for specificity examination.

**k** Dot blot analysis of antibodies specific to methylated RIG‑I at K18 or K146 with K18 or K146 unmodified (free) and mono-methylated (me) peptides as indicated.

**l** Methylated RIG-I at K18 or K146 were examined by the specific antibodies in the precipitates by total RIG-I antibody from primary hepatocytes upon IL-6 stimulation.

**m** Construction of RIG-I K18M+K146M or K18A+K146A mutant mice.

**n** IL-6-induced STAT3 phosphorylation was evaluated in isolated primary hepatocytes from wildtype, RIG-I K18M+K146M or K18A+K146A mutant mice.

Data are shown as photographs from one representative of three independent experiments.

**
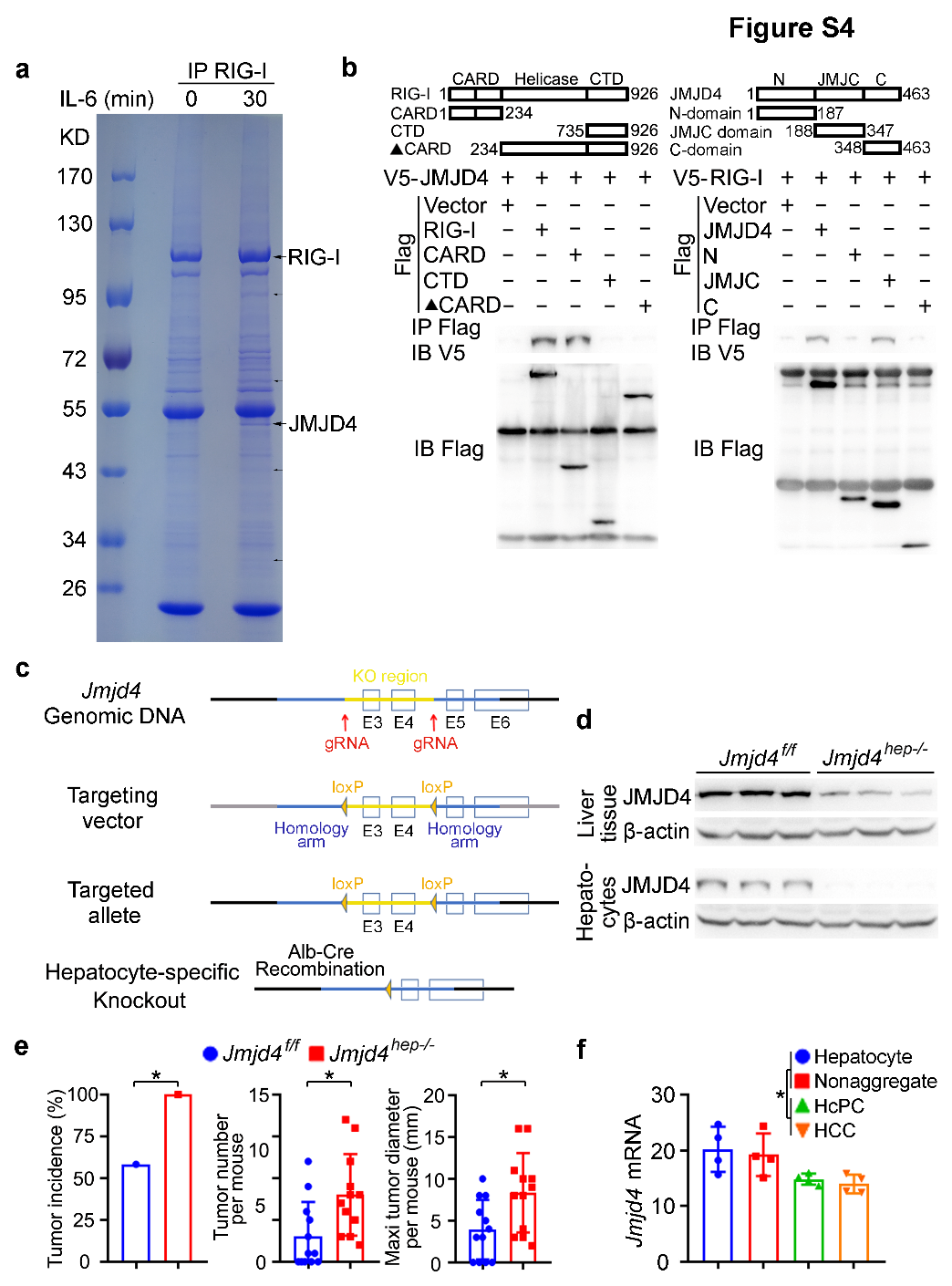
**

**Figure S4** JMJD4 associates and demethylates RIG-I to suppress DEN-induced hepatocarcinogenesis.

**a** PAGE gel resolution of immunoprecipitated RIG-I and its associated proteins from HHL5 hepatocyte cell line treated with IL-6 for 30 minutes. Different bands were analyzed by MS. Arrow indicates the band of the protein detected by MS.

**b** V5-tagged JMJD4 and Flag-tagged RIG-I truncates, V5‑tagged RIG-I and Flag‑tagged JMJD4 truncates as indicated were transfected into HHL5 hepatocyte cell line, and JMJD4-RIG‑I association was tested by immunoprecipitation.

**c** Construction of *Jmjd4^hep-/-^* mice.

**d** JMJD4 expression in liver tissues and isolated hepatocytes from *Jmjd4^hep-/-^* mice was confirmed by Western blot.

**e** Tumor incidence (chi-square test), number and maximum diameter (unpaired *t*-test) of DEN plus CCl_4_-induced HCC in male *Jmjd4^f/f^* and *Jmjd4^hep‑/-^* mice were analyzed (*n*=12).

**f** *Jmjd4* mRNA in isolated normal hepatocytes, nonaggregated hepatocytes and HcPCs from male mice five months post DEN injection, and established HCC cells eight months post DEN injection was examined by qRT-PCR (*n*=4, one-way ANOVA and Tukey’s multiple comparisons test).

Data are shown as mean ± s.d. or photographs from one representative of three independent experiments. **P*<0.05.

**
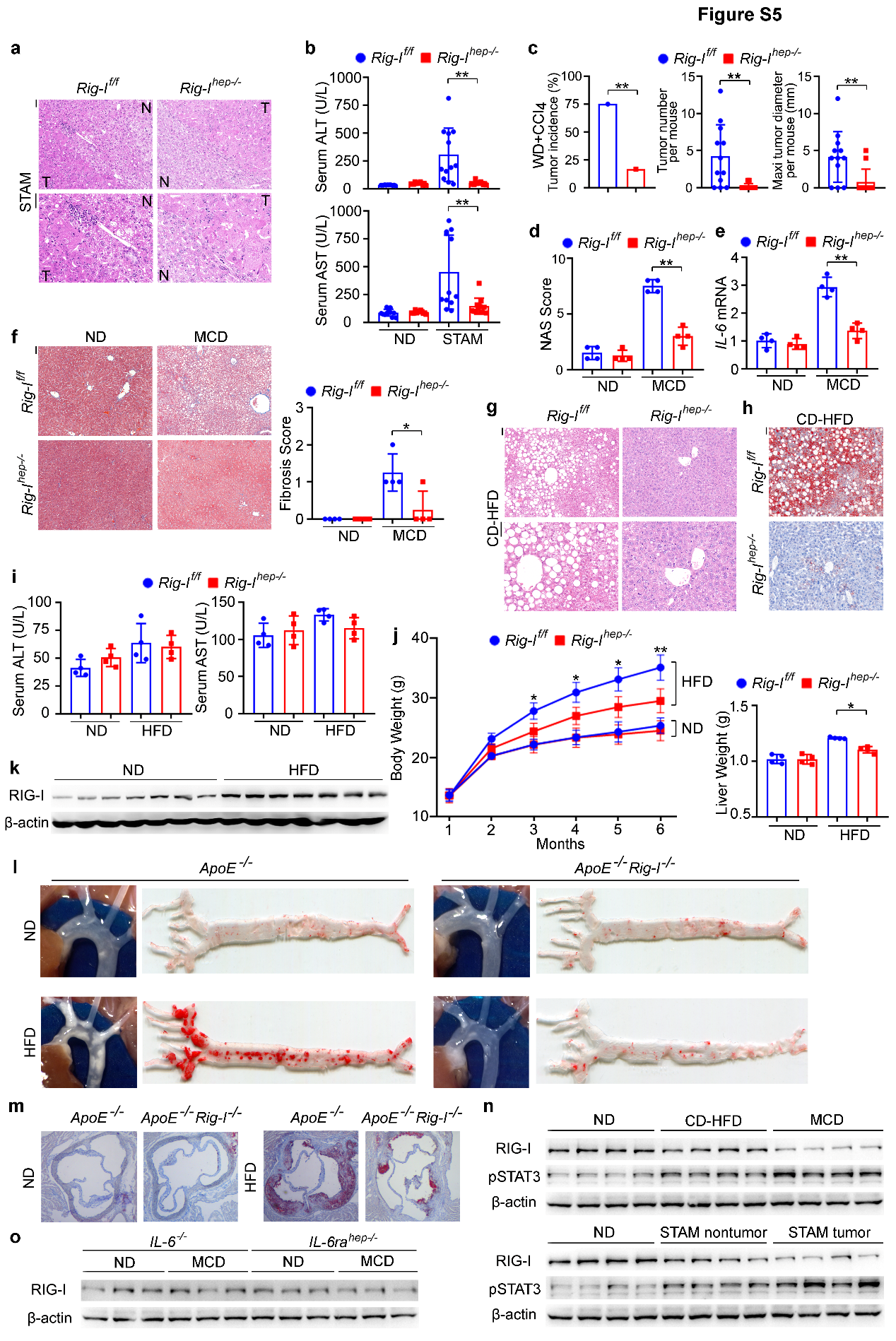
**

**Figure S5** Hepatocyte-specific RIG-I deficiency abolishes steatosis, and the following NASH, and NASH-induced hepatocarcinogenesis.

**a** HE staining was analyzed in STAM tumor tissues of male *Rig-I^f/f^* and *Rig-I^hep-/-^* mice (T, tumor; N, nontumor). Scale bars: 20 μm.

**b** Serum ALT and AST were examined in STAM model of male *Rig-I^f/f^* and *Rig-I^hep‑/-^* mice (*n*=4, unpaired *t*-test).

**c** Tumor incidence (chi-square test), number and maximum diameter (unpaired *t*-test) of WD+CCl_4_-induced HCC in male *Rig-I^f/f^* and *Rig-I^hep-/-^* mice were analyzed (*n*=12).

**d** NAS score of HE staining was analyzed in liver tissues of MCD model in male *Rig‑I^f/f^* and *Rig-I^hep-/-^* mice (*n*=4, unpaired *t*-test).

**e** *IL-6* mRNA level in liver tissues of MCD model in male *Rig‑I^f/f^* and *Rig-I^hep-/-^* mice was examined using qRT-PCR (*n*=4, unpaired *t*-test).

**f** Masson staining and fibrosis score were analyzed as indicated in liver tissues of MCD model in male *Rig‑I^f/f^* and *Rig-I^hep-/-^* mice (*n*=4, unpaired *t*-test). Scale bars: 20 μm.

**g,h** HE (**g**) and oil red O (**h**) staining were analyzed in liver tissues of CD-HFD model in male *Rig‑I^f/f^* and *Rig-I^hep-/-^* mice. Scale bars: 20 μm.

**i** Serum ALT and AST were analyzed in HFD-treated male *Rig-I^f/f^* and *Rig-I^hep‑/-^* mice (*n*=4).

**j** Body and liver weight of HFD-treated male *Rig-I^f/f^* and *Rig-I^hep‑/-^* mice (*n*=4).

**k** RIG-I expression in liver tissues of HFD-treated male mice were examined by Western blot.

**l,m** Representative aortic arch and oil red O staining were shown as indicated in *ApoE^‑/-^* and *ApoE^-/-^Rig-I^-/-^* mice treated with HFD and 1.25% cholesterol for 12 weeks.

**n** RIG-I expression and STAT3 phosphorylation in liver tissues of CD-HFD or MCD models, and tissues of STAM nontumor or tumor were examined by Western blot as indicated.

**o** RIG-I expression in liver tissues of MCD model in IL-6 knockout or hepatic IL-6 receptor knockout mice was examined by Western blot as indicated.

Data are shown as mean ± s.d. or photographs from one representative of three independent experiments. **P*<0.05, ***P*<0.01.

**
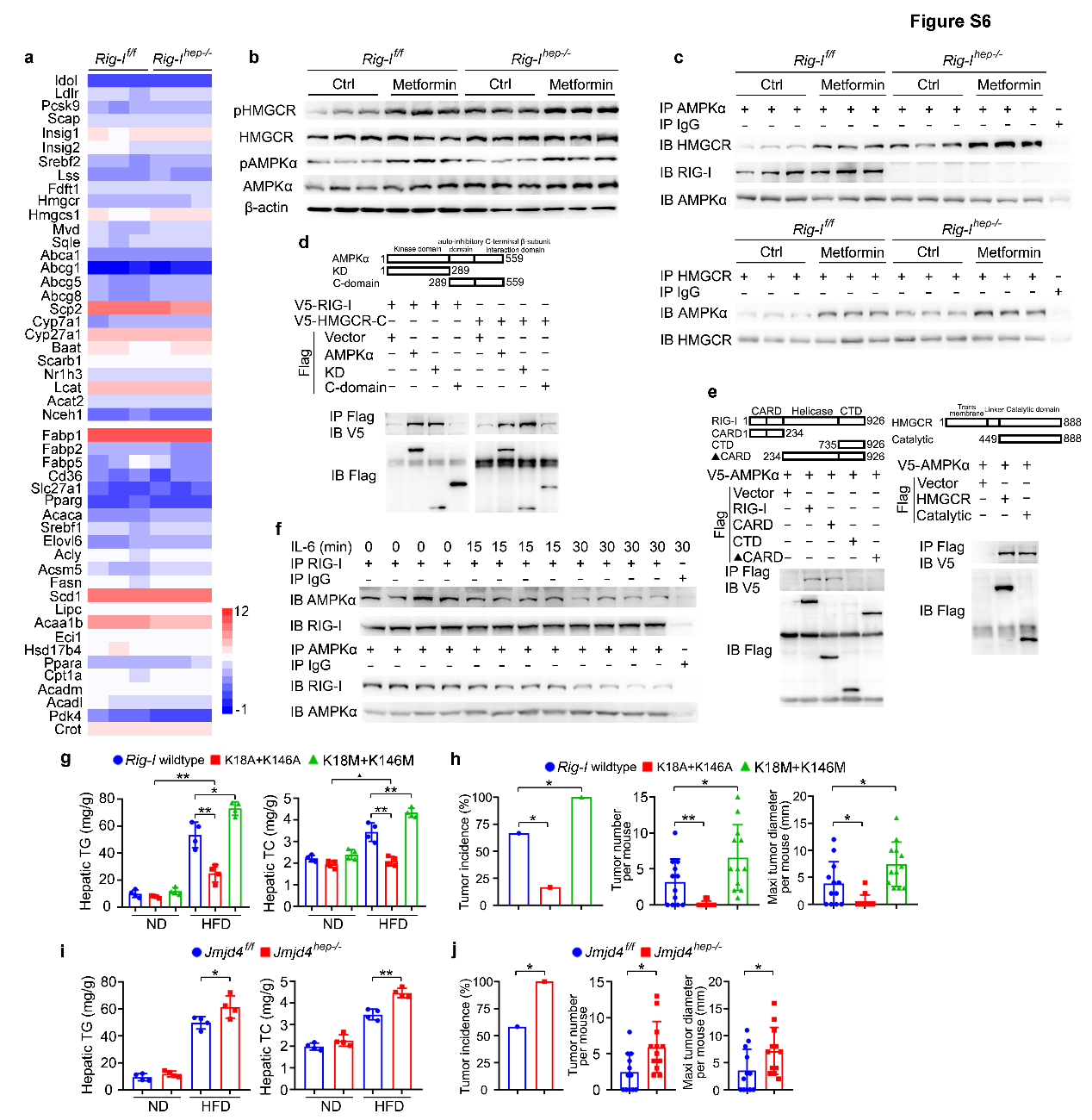
**

**Figure S6** Methylated RIG-I associates AMPKα to inhibit HMGCR phosphorylation and enhance cholesterol synthesis.

**a** mRNA levels of genes for lipid uptake, synthesis, transport, and excretion were analyzed using RNA-seq in the liver tissues of male *Rig-I^f/f^* and *Rig‑I^hep‑/-^* mice (*n*=3).

**b** HMGCR, AMPKα, and their phosphorylation were examined in the liver tissues of male *Rig-I^f/f^* and *Rig‑I^hep‑/-^* mice treated with Metformin.

**c** The AMPKα-HMGCR interaction and AMPKα-RIG-I interaction upon Metformin treatment were evaluated using immunoprecipitation in the liver tissues from male *Rig‑I^f/f^* and *Rig‑I^hep‑/-^* mice.

**d** V5-tagged RIG-I, V5‑tagged‑HMGCR-C, and Flag-tagged AMPKα truncates as indicated were transfected into HHL5 hepatocyte cell line, and AMPKα-RIG-I and AMPKα-HMGCR association were tested by immunoprecipitation.

**e** V5-tagged AMPKα, Flag‑tagged‑RIG-I and HMGCR truncates as indicated were transfected into HHL5 hepatocyte cell line, and AMPKα-RIG-I and AMPKα-HMGCR association were tested by immunoprecipitation.

**f** The association between AMPKα and RIG-I modulated by IL-6 was examined by immunoprecipitation in the liver tissues from male mice upon IL-6 stimulation.

**g** Hepatic TG and TC were examined in HFD-treated male wildtype, RIG-I K18M+K146M or K18A+K146A mutant mice (*n*=4, unpaired *t*-test).

**h** Tumor incidence (chi-square test), number and maximum diameter (unpaired *t*-test) of STAM HCC in male wildtype, RIG-I K18M+K146M or K18A+K146A mutant mice were analyzed (*n*=12).

**i** Hepatic TG and TC were analyzed in HFD-treated male *Jmjd4^f/f^* and *Jmjd4^hep‑/-^* mice (*n*=4, unpaired *t*-test).

**j** Tumor incidence (chi-square test), number and maximum diameter (unpaired *t*-test) of STAM HCC in male *Jmjd4^f/f^* and *Jmjd4^hep‑/-^* mice were analyzed (*n*=12).

Data are shown as mean ± s.d. or photographs from one representative of three independent experiments, or heatmap as indicated. **P*<0.05, ***P*<0.01, ^▲^*P*>0.05.

**
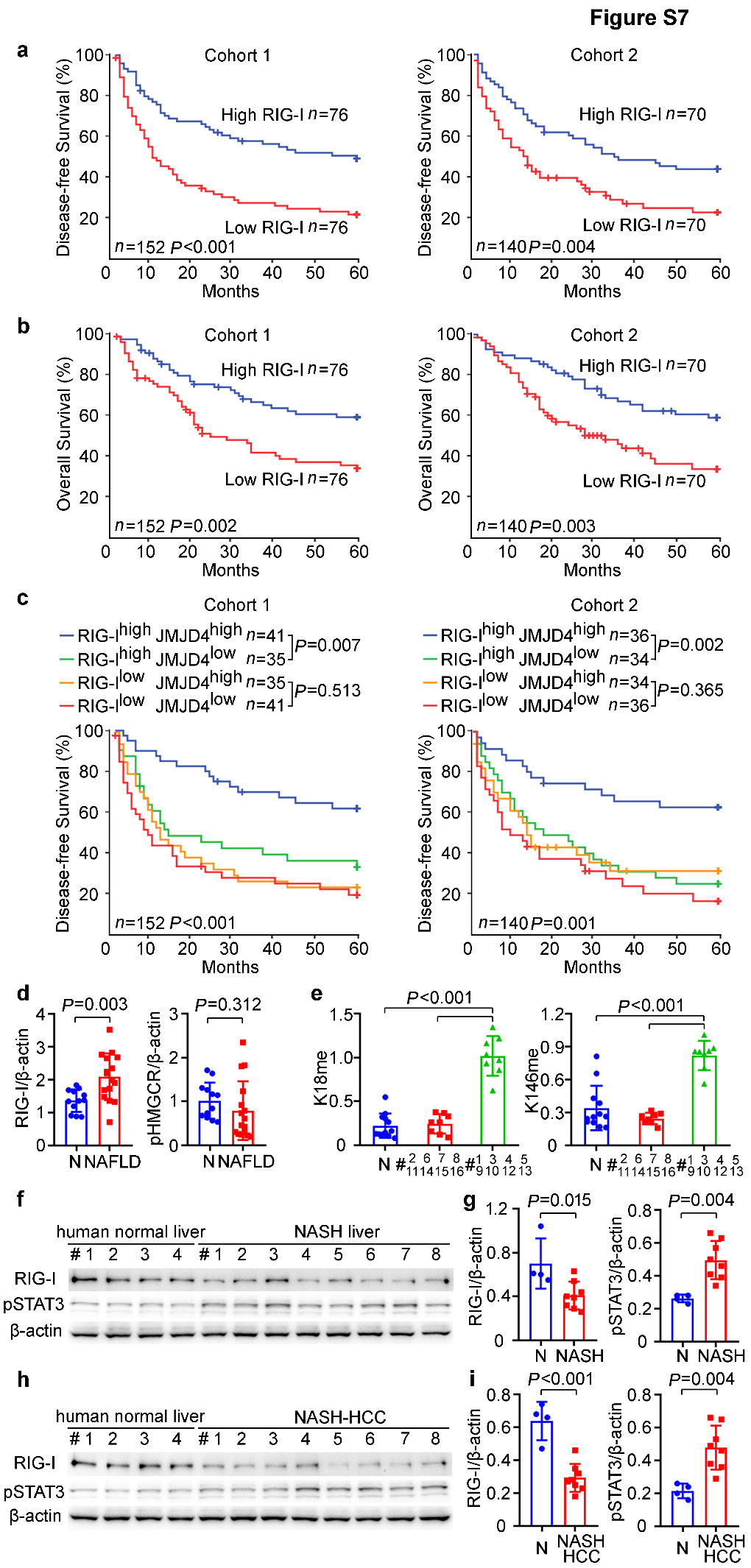
**

**Figure S7** RIG-I expression and methylation may be correlated to human hepatocarcinogenesis, prognosis, and NAFLD progression.

**a**,**b** Kaplan‑Meier survival curves of disease-free survival (**a**) and overall survival (**b**) based on dichotomized *RIG-I* mRNA expression in HCC tissues of Cohort 1 and 2. The median levels of *RIG-I* expression in each cohort were used as the cutoff, with log‑rank test for significance.

**c** Kaplan-Meier survival curves of disease-free survival based on dichotomized *RIG-I* and *JMJD4* mRNA expression in HCC tissues of Cohort 1 and 2. The median levels of *RIG-I* and *JMJD4* expression in each cohort were used as the cutoff, with log-rank test for significance.

**d** Quantified RIG-I protein level and HMGCR phosphorylation level in human normal liver tissues (N, *n*=12) and NAFLD tissues (*n*=16, unpaired *t*-test) were shown.

**e** Quantified levels of methylated RIG-I at K18 or K146 in human normal liver tissues (N, *n*=12) and the indicated NAFLD tissues (*n*=8, unpaired *t*‑test) were shown.

**f,g** RIG-I expression and STAT3 phosphorylation (**f**) in human normal liver tissues (*n*=4) and NASH tissues (*n*=8) were analyzed by Western blot, with their quantification shown as indicated (unpaired *t*-test) (**g**).

**h,i** RIG-I expression and STAT3 phosphorylation (**h**) in human normal liver tissues (*n*=4) and NASH-HCC tissues (*n*=8) were analyzed by Western blot, with their quantification shown as indicated (unpaired *t*-test) (**i**).

Data are shown as mean ± s.d., survival curves, or photographs as indicated.
